# The long non-coding RNA GAS5 differentially regulates cell cycle arrest and apoptosis through activation of BRCA1 and p53 in human neuroblastoma

**DOI:** 10.18632/oncotarget.14244

**Published:** 2016-12-27

**Authors:** Joseph Mazar, Amy Rosado, John Shelley, John Marchica, Tamarah J Westmoreland

**Affiliations:** ^1^ Department of Research, Nemours Children's Hospital, Orlando, FL 32827, USA; ^2^ Sanford Burnham Prebys Medical Discovery Institute, Orlando, FL 32827, USA

**Keywords:** long non-coding RNA, neuroblastoma, cancer, GAS5, p53

## Abstract

The long non-coding RNA GAS5 has been shown to modulate cancer proliferation in numerous human cancer systems and has been correlated with successful patient outcome. Our examination of GAS5 in neuroblastoma has revealed robust expression in both MYCN-amplified and non-amplified cell lines. Knockdown of GAS5 *In vitro* resulted in defects in cell proliferation, apoptosis, and induced cell cycle arrest. Further analysis of GAS5 clones revealed multiple novel splice variants, two of which inversely modulated with MYCN status. Complementation studies of the variants post-knockdown of GAS5 indicated alternate phenotypes, with one variant (FL) considerably enhancing cell proliferation by rescuing cell cycle arrest and the other (C2) driving apoptosis, suggesting a unique role for each in neuroblastoma cancer physiology. Global sequencing and ELISA arrays revealed that the loss of GAS5 induced p53, BRCA1, and GADD45A, which appeared to modulate cell cycle arrest in concert. Complementation with only the FL GAS5 clone could rescue cell cycle arrest, stabilizing HDM2, and leading to the loss of p53. Together, these data offer novel therapeutic targets in the form of lncRNA splice variants for separate challenges against cancer growth and cell death.

## INTRODUCTION

Neuroblastoma is one of the most common extra cranial tumors of childhood, and the most commonly diagnosed malignancy in infants [1–3]. With challenging heterogeneity within the tumor subpopulation, prognostic factors for survival include age at diagnosis, tumor grade, tumor site, histology, and amplification of the v-myc avian myelocytomatosis viral oncogene neuroblastoma derived homolog *(MYCN)* gene [4, 5]. *MYCN* amplification is one of the most important markers that correlate with advanced disease and poor survival with approximately 20% - 25% of patients containing the *MYCN* amplification [6, 7]. Over the last 20 years, there has not been great improvement in the overall survival of children with MYCN-amplified neuroblastoma. As a result, it is imperative that we focus on the underlying genetic changes that are occurring in this high-risk group.

To this end, we are investigating the molecular events surrounding neuroblastoma, with emphasis on long non-coding RNAs (lncRNAs) and their role in neuroblastoma. Long non-coding RNAs are RNA molecules larger than 200 nucleotides which do not translate into proteins [8]. Originally thought to be “junk” RNA, there is increasing evidence that lncRNAs are involved in a wide range of biological functions, including cell differentiation, proliferation, and apoptosis, among others [2, 9].

Growth Arrest-Specific 5 (GAS5) is a lncRNA first isolated from NIH 3T3 mouse fibroblasts. GAS5 was named based on the finding that, after serum starvation [10] or rapamycin-induced cell cycle arrest [11], expression levels increased. GAS5 consists of 12 exons and 10 box C/D snoRNAs (Small nucleolar RNAs), as well as a conserved 5′-terminal oligopyrimidine tract (5′ TOP) [11], with at least 29 documented splice variants. Modulation of certain GAS5 splice variants has been reported to induce growth arrest and apoptosis in some human cell lines [12], but the full extent of the functional characteristics of these variants have yet to be studied. Furthermore, GAS5 expression has been shown to be decreased in other advanced tumors, such as breast cancer [13, 14], bladder cancer [15], gastric cancer [16], and non-small-cell lung cancer [17].

Our analysis of GAS5 in neuroblastoma indicates it is expressed in both MYCN-amplified and non-amplified cell lines. Knockdown of GAS5 in neuroblastoma cell lines revealed defects in cell proliferation, apoptosis, and cell cycle arrest. Further analysis of sequenced GAS5 clones revealed multiple novel splice variants, two of which appear to modulate expression between MYCN-amplified and non-amplified cells. These two variants, dubbed “Full-Length” (FL) and “Clone 2” (C2), were capable of complementing defects seen due to general loss of GAS5, but the FL variant further enhanced cell proliferation and rescued cell cycle arrest, whereas the C2 variant had only a minimal effect on apoptosis. Analysis of global transcriptional changes due to the loss of GAS5 revealed an induction of p53 which appeared to be responsible for the initiation of cell cycle arrest. Further analysis revealed increased phosphorylation of p53, as well as BRCA1, both of which appeared to contribute to induction of arrest through activation of GADD45A. Knockdown of either BRCA1 or GADD45A could rescue cell arrest, though loss of p53 greatly enhanced apoptosis as well. Knockdown of GAS5, followed by complementation with the GAS5 FL variant, but not the C2 variant, rescued cell cycle arrest by stabilization of HDM2, leading to the loss of p53. Together, these data indicate that GAS5 expression has a significant impact on neuroblastoma cell biology and differential expression of its splice variants could act to regulate physiological priorities toward cell proliferation or regulation of apoptosis.

## RESULTS

### lncRNA GAS5 is highly expressed in both MYCN-amplified and non-amplified neuroblastoma cell lines

**Figure 1:**
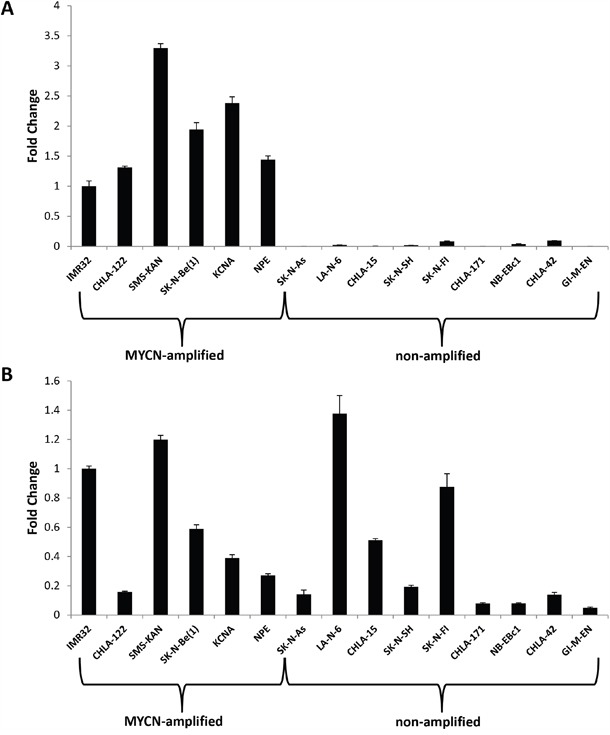
Expression of MYCN and the lncRNA GAS5 in human neuroblastoma **A**. MYCN and **B**. GAS5 expression in neuroblastoma cell lines as measured by qRT-PCR. MYCN expression is confirmed as amplified in IMR-32, CHLA-122, SMS-KAN, SK-N-Be(1), KCNA, and NPE cell lines. GAS5 is highly expressed in both MYCN-amplified and non-amplified cell lines. The Ct value of each sample was normalized to the Ct value of GAPDH, and the relative expression was calculated by normalizing to IMR-32 cells by calculating the ΔΔCt method. Data are expressed as means +/- SD from three biological replicates for each sample.

Expression of the lncRNA GAS5 has been shown to have a physiological impact on numerous human cancer systems [12–16]. In order to determine if there is a correlation between MYCN and GAS5 expression levels in neuroblastoma, 15 neuroblastoma cell lines were screened (6 MYCN-amplified and 9 non-amplified) for both MYCN and GAS5 expression by qRT-PCR, normalized to GAPDH. GAS5 was measured specifically from Exons 11 and 12, allowing for the measurement of the broadest possible combination of variants (23 out of 29 possible splice variants). Figure 1A confirms MYCN amplification compared to the non-amplified cell lines, whereas Figure 1B reveals GAS5 expression in these correlating cell samples. The results confirm an enormous contrast in MYCN expression between amplified and non-amplified lines (with the differences as much as 300-fold), whereas GAS5 expression varied, though considerably less (by no more than 20-fold). However, no correlation could be determined between MYCN amplification (or lack thereof) and GAS5 expression.

### Discovery of differentially expressed GAS5 splice variants in MYCN-amplified vs. non-amplified neuroblastoma cells

**Figure 2:**
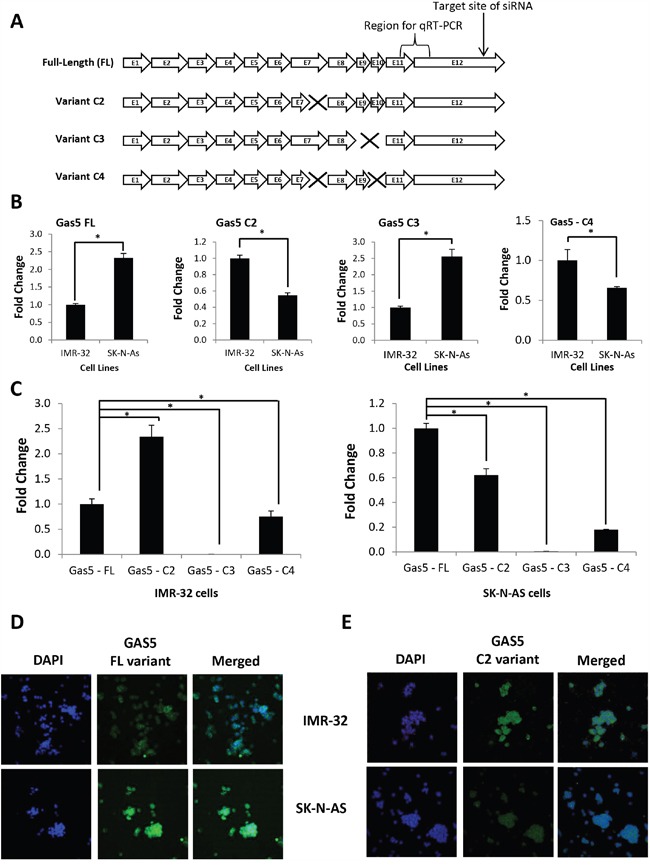
Discovery of GAS5 splice variants differentially expressed in MYCN-amplified vs. non-MYCN-amplified neuroblastoma cells **A**. Schematic indicating the layout of exons present in GAS5, including “Full-Length” (FL) and novel splice variants “Clone 2” (C2), “Clone 3” (C3), and “Clone 4” (C4). Exons or partial exons “missing” or deleted from novel splice variants indicted by “X”. Schematic also indicates the location for the initial quantitation of GAS5 by qRT-PCR primers and the target site of the GAS5 siRNA used in these experiments. **B**. Comparison of the expression of individual GAS5 splice variants as measured between IMR-32 and SK-N-AS cells by qRT-PCR. Comparisons performed for FL, C2, C3, and C4 variants using splice-specific primer sets. **C**. Direct comparison of the expression of GAS5 splice variants as measured within IMR-32 or SK-N-AS cells by qRT-PCR. **D** and **E**. Expression of individual GAS5 splice variants FL and C2 by RNA fluorescence *in situ hybridization* staining of IMR-32 and SK-N-AS cells. GAS5 staining is in green (FITC) and nuclei are stained in blue (DAPI). Samples are also shown together (merged). All qRT-PCR samples normalized to GAPDH and performed in triplicate from three biological replicates. * p < 0.05, Student's t-test.

In order to better characterize GAS5 in neuroblastoma cells, we decided to clone the lncRNA. Primers were designed (variant – 001, ensembl.org) and GAS5 was cloned from cDNA acquired from IMR-32 cell total RNA. Sequencing of these clones revealed multiple splice variants (Figure 2A), which in itself was not surprising given that over two dozen splice variants for GAS5 have already been verified [11]. However, in addition to variant – 001 (hereafter referred to as “Full-Length” or “FL”), three other variants were discovered, each of which are novel products so far unreported (hereafter referred to as “Clone #2” or “C2”, “Clone #3” or “C3”, and “Clone #4” or “C4”). The C2 variant differed from the FL product due to a loss of 39 base pairs from the end of exon 7 (a 77 bp exon). The C3 variant contained neither exons 9 nor 10. The C4 variant had both a complete loss of exon 10 and the identical loss of 39 bp from Exon 7 seen in C2. Primer sets were designed to specifically quantify each variant and qRT-PCR was performed to measure the expression of each variant in the two cell lines (Figure 2B & Supplementary Figure 1). The results indicated that expression of both the FL and C3 splice variants were significantly higher in SK-N-AS cells (225% and 250%, respectively), whereas expression of the C2 and C4 splice variants were higher in IMR-32 cells (200% and 165%, respectively). A further comparison of splice variant content within IMR-32 cells revealed that whereas the FL clone is well expressed, the best expressed product was, in fact, the C2 variant (230% of FL expression) (Figure 2C). Interestingly, when individual splice variant profiling was performed in SK-N-AS cells, the FL product was the highest expressed of the GAS5 products, with the C2 variant at only 60% of FL levels (Figure 2C). Since the C3 variant appeared to be by comparison poorly expressed in both cell lines and the C4 variant tracked below both the FL and C2 products in both systems as well, we focused our continuing efforts on the FL and C2 variants. Profiling of the FL and C2 variants was also performed in 15 original neuroblastoma cell lines as well (Supplementary Figure 2A & 2B). In order to confirm the native expression levels of the FL and C2 variants, probes were constructed, and expression was examined by Fluorescence *In Situ* Hybridization (FISH). Labeling of the FL variant confirmed significantly higher expression in SK-N-AS cells than in IMR-32 cells (Figure 2D). Likewise, labeling of the C2 variant indicated more robust expression in IMR-32 cells than SK-N-AS cells (Figure 2E). These results led us to question whether modulation of the FL and C2 variants might have biological relevance. Given that IMR-32 cells are a MYCN amplified neuroblastoma cell line, and SK-N-AS cells are a non-MYCN amplified cell line, we compared splice variant content within other MYCN-amplified cell lines (CHLA-122 and SMS-KAN) and non-MYCN-amplified cell lines (LA-N-6 and CHLA-15). The results indicated a comparable pattern, with the C2 variant significantly higher expressed in both CHLA-122 and SMS-KAN cells (at 240% and 230% of FL, respectively), whereas the C2 variant was significantly lower expressed in both LA-N-6 and CHLA-15 cells (C2 only 65% and 67% of FL, respectively) (Supplementary Figure 3A & 3B). Together, these data indicate that a pattern of differential GAS5 splice variant expression may be present between MYCN-amplified and non-amplified neuroblastoma cell lines.

### Loss of GAS5 in MYCN-amplified neuroblastoma cells decreases cell proliferation and apoptosis, as well as inducing cell cycle arrest

**Figure 3:**
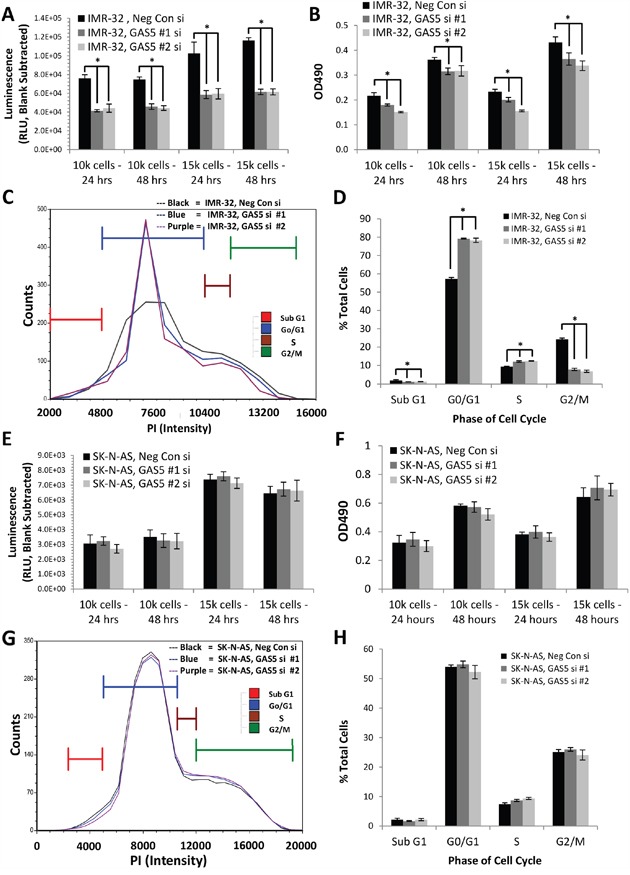
Effect of GAS5 knockdown on human neuroblastoma cells **A**. Loss of GAS5 in IMR-32 cells decreased apoptosis rate at both 24 and 48 hr time points. **B**. Loss of GAS5 in IMR-32 cells decreased cell proliferation rate at both 24 and 48 hr time points. **C** and **D**. Knockdown of GAS5 in IMR-32 cells increased the content of G0/G1 cells and decreased the content of G2/M phase cells, inducing cell cycle arrest, as measured by propidium iodide staining. **E**. Loss of GAS5 in SK-N-AS cells caused no significant changes in apoptosis rate at either 24 or 48 hr time points. **F**. Loss of GAS5 in SK-N-AS cells had no detectable effect on cell proliferation rate at both 24 and 48 hr time points. **G** and **H**. Knockdown of GAS5 in SK-N-AS cells yielded no significant changes in either G0/G1 or G2/M phase cells as measured by propidium iodide staining. All experiments were performed in triplicate. * p < 0.05, Student's t-test.

In order to determine the function of GAS5 expression in human neuroblastoma, both MYCN-amplified (IMR-32) and non-amplified (SK-N-AS) cell lines were transfected with two separate GAS5 siRNAs. qRT-PCR confirmed knockdown by as much as 70% in both cell lines (Supplementary Figure 4A & 4B) with either siRNA compared to Negative Control siRNA. The cells were then examined for changes in cell biological processes. Measurement of apoptosis in IMR-32 cells revealed that reduction of GAS5 by either siRNA significantly decreased the rate of apoptosis (by as much as 60% at 24 hours) compared to Negative Control siRNA (Figure 3A). Likewise, measurements of cell proliferation (Figure 3B) indicated a decrease in viability (by as much as 30% at 24 hours) compared to Negative Control siRNA. Propidium iodide labeling of GAS5 knocked-down IMR-32 cells indicated a significant increase in cell population at G0/G1 (from 56% to 79% of total cells), an increase in cells at S phase (from 9% to 12%), but a dramatic decrease in G2/M phase cells (dropping from 24% to 7%), suggesting that the cells were undergoing cell cycle arrest (Figure 3C & 3D). An examination of SK-N-AS cells, however, gave significantly different results. Measurements of apoptosis in GAS5 knocked-down cells revealed marginal changes compared to Negative Control siRNA, at either 24 or 48 hours. Cell proliferation rate revealed no statistically significant change at either time point and propidium iodide labeling indicated changes to G0/G1, S, and G2/M phases that were no greater than 5% in any category (Figure 3E–3H).

Given that a MYCN-amplified cell line showed significant cellular changes to the loss of GAS5 and a non-MYCN-amplified cell line did not, we decided to examine a separate non-amplified cell line, LA-N-6, to determine if MYCN amplification may be essential to this phenotype. Surprisingly, loss of GAS5 in LA-N-6 cells revealed significant decreases in apoptosis (by as much as 40% by 24 hours) and cell viability (by as much as 25% by 24 hours) (Supplementary Figure 4C, 5A & 5B). In addition, propidium iodide staining revealed increases in G0/G1 (from 65% to 80% of total cells) and decreases in G2/M (dropping from 24% to 9%), indicating cell cycle arrest (Supplementary Figure 5C & 5D). These data suggest that the impact of the loss of GAS5 in neuroblastoma cells can vary depending upon the cell type and may not be dependent upon MYCN-amplification.

In order to determine if MYCN-amplification could affect GAS5 expression, we performed an siRNA knock-down of MYCN in IMR-32 cells (Supplementary Figure 6A-6D). The results indicated that the loss of MYCN does have some effect on GAS5 expression, although limited (a 50% loss of MYCN yielded only a 20% drop in GAS5 expression), confirming that MYCN is not required for GAS5 expression, though may play a role in its transcription. However, those cells that do respond to a loss of GAS5 appear to enter a state of cell cycle arrest, one which precludes apoptosis, suggesting possible down-stream regulators which control both of these processes.

### The GAS5 splice variants FL and C2 differentially modulate cellular phenotypes

**Figure 4:**
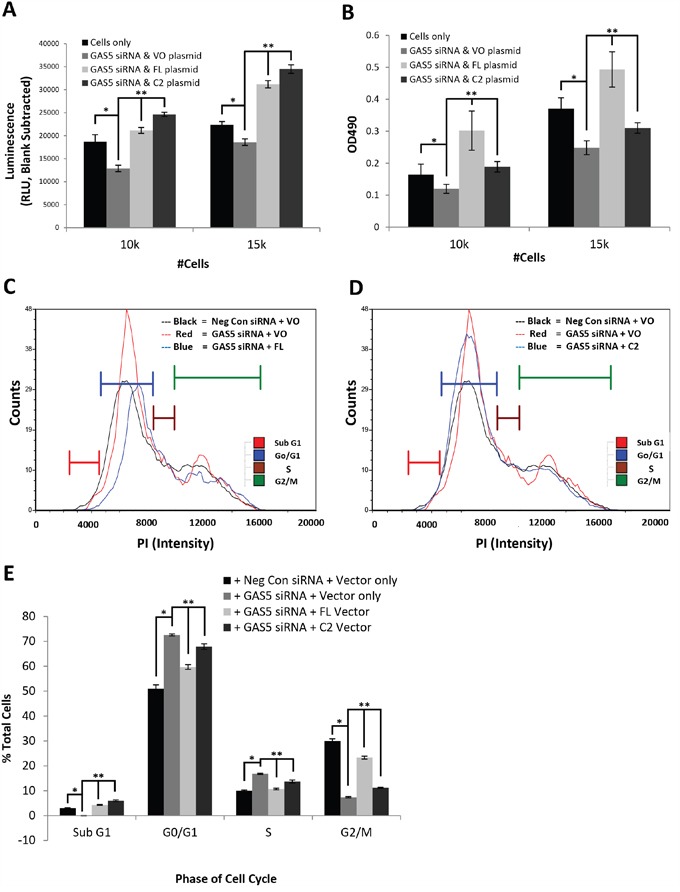
Differential modulations of cellular functions by GAS5 splice variants, FL and C2, in IMR-32 cells **A**. Measurement of apoptosis rate in IMR-32 cells knocked-down for GAS5 and complemented with individual variants FL or C2. Both variants can compensate for a decrease in apoptosis due to global loss of GAS5. However, the C2 variant increases apoptosis rate slightly more than FL. **B**. Measurement of cell viability in IMR-32 cells knocked-down for GAS5 and complemented with individual variants FL or C2. Both variants can compensate for viability loss due to global decrease in GAS5. However, the FL variant increases proliferation considerably more than C2. **C-E**. Measurement of cell cycle populations after knockdown of GAS5 followed by complementation with C) the FL splice variant or D) the C2 splice variant in IMR-32 cells. Complementation of the FL variant appears to rescue cell cycle arrest induced by loss of GAS5, whereas the C2 variant does not appear to do so. All experiments were performed in triplicate. * and ** p < 0.05, Student's t-test.

In order to determine if the FL and C2 GAS5 splice variants had differential effects on cell physiology, we decided to knockdown GAS5 by siRNA and complement by exogenous expression of the individual variants. Since the loss of GAS5 gave significantly more pronounced physiological effects in IMR-32 cells than SK-N-AS cells, we decided to examine the impact of the individual splice variants in this cell line first. Cells were transfected with two separate GAS5 siRNAs and the efficiency of knockdown was confirmed for both FL and C2 variants by qRT-PCR (Supplementary Figure 7A-7C). In order to examine the individual effects of the variants, both the FL and C2 genes were sub-cloned to construct exogenous expression vectors (pcDNA6/GAS5 FL and pcDNA6/GAS5 C2). IMR-32 cells were then transfected with GAS5 siRNA and complemented with either FL, C2, or the parental vector (vector only). An examination of apoptosis activity confirmed that both variants could compensate for the loss of apoptosis (Figure 4A), but C2 increased apoptosis more so than FL (30-40% vs. 20-25% compared to parental vector, respectively). The results of a cell proliferation assay also indicated that both variants could individually compensate for the loss of cell viability (Figure 4B). However, viability was additionally increased by as much as 50% as a result of the FL variant, suggesting that in IMR-32 cells, this variant was considerably more competent to drive cell proliferation than the C2 variant. Together, these experiments indicate that compensation with individual variants can recover apoptosis rate, but expression of the FL variant greatly increases cell proliferation, without a commensurate increase in apoptosis, suggesting that this variant would be the better choice for use in faster growing cells.

In order to measure changes in cell cycle activity, propidium iodide labeling was performed, corroborating the previous changes seen in IMR-32 cells upon loss of GAS5. However, complementation with the FL variant significantly corrected cell cycle changes (Figure 4C & 4E, and Supplementary Figure 8A), decreasing the G0/G1 population (from 72% to 60%) and S phase population (from 17% to 11%), while increasing the G2/M phase population (rising from 7% to 23%). Even the Sub G0 population increased by 4% (corroborating the results seen in Figure 4A). The overall effect of these changes revealed a pattern more comparable with parental vector conditions. These were not the results seen, however, by complementation with the C2 splice variant (Figure 4D & 4E, and Supplementary Figure 8B). By comparison, the C2 variant caused only mild decreases in the G0/G1 population (from 72% to 68%) and S phase population (from 17% to 14%), while only slightly increasing the G2/M phase population (rising from 7% to 11%). The largest overall changes were actually seen in the Sub G0 population, which increased by 6% (again corroborating the results seen in Figure 4A), leaving the apoptosis rate significantly higher than parental vector cells. These results suggest that FL compensates for much of the changes seen in IMR-32 cells due to the loss of GAS5, whereas the C2 variant only poorly does so, indicating that the capabilities of these individual splice variants vary greatly, even in the same cell system.

### The loss of GAS5 in neuroblastoma cells leads to modulation of the p53 cell cycle regulator

**Figure 5:**
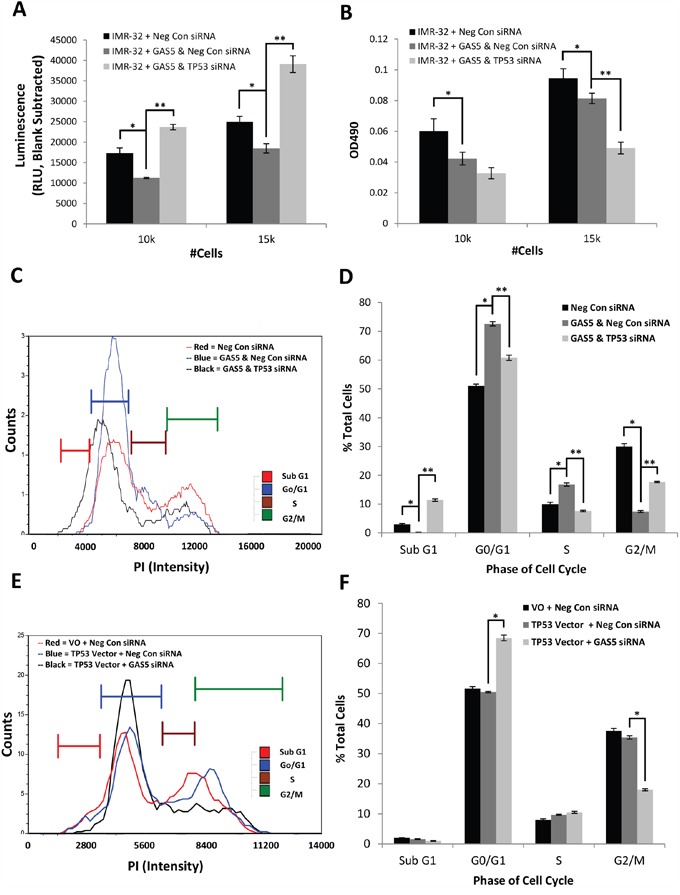
Effect of p53 Knockdown in Concert with loss of GAS5 Measurement of cell biology assays in IMR-32 cells coordinately knocked-down for both p53 and GAS5: **A**. Measurement of Apoptosis reveals that additional loss of p53 rescues the drop in apoptosis from GAS5 alone, **B**. Measurement of cell viability indicates further loss of cell viability when p53 is additionally knocked-down, **C** and **D**. Measurement of cell cycle populations after additional loss of p53 reveals significant rescue of cell cycle arrest at the cost of a dramatic increase in apoptosis. **E** and **F**. Compensation of the exogenous expression of wild type p53 in SK-N-AS cells in concert with GAS5 knockdown induces a state of cell cycle arrest. All experiments were performed in triplicate. * and ** p < 0.05, Student's t-test.

In order to determine a possible mechanism of action for the cellular changes seen in neuroblastoma cells after the loss of GAS5, total RNA was acquired from IMR-32 cells 48 hours after transfection with either Negative Control siRNA or GAS5 siRNA and Next-Gen sequencing (RNA-Seq) was performed ( https://www.ncbi.nlm.nih.gov/geo/query/acc.cgi?acc=GSE90523). Global changes in the transcriptome were examined and clusters were assessed for the greatest changes in predicted biological function (Supplementary Figure 9A). The results revealed that the top global networks modulated by reduction of GAS5 involved changes in hereditary, metabolic, and neurological diseases. The top diseases or biological functions associated with these changes correlated most likely with molecules involved in either cancer or organismal injury, specifically related to cell growth or proliferation. An examination of this specific cluster of transcripts revealed a number of possible genes whose functions were related to cell growth including Tumor Protein 53 (TP53 or p53), whose steady-state expression was significantly increased. p53 is a well-known tumor suppressor often associated with induced cell death through apoptosis, though it also functions to induce cell cycle arrest [18–21]. Quantification of p53 by qRT-PCR corroborated the RNA-Seq data with an ~70% increase in expression (Supplementary Figure 9B). Given that knockdown of GAS5 in IMR-32 cells appeared to induce cell cycle arrest and this appeared to correlate with induction of the p53 transcript, we decided to pursue this gene as a possible explanation of the cellular phenotypes we had witnessed. In order to determine if induction of p53 was relevant to the GAS5 knockdown phenotypes, p53 siRNA was co-transfected with GAS5 siRNA and compared to co-transfected Negative control siRNA. qRT-PCR of GAS5 levels confirmed loss in transfected cells (Supplementary Figure 10A), indicating that the siRNAs used were successful. Measurements of apoptosis rate revealed that the decrease seen due to the loss of GAS5 was more than compensated for when p53 was knocked-down, with apoptosis increasing above wild type levels (Figure 5A). This suggested that p53 induction might have been responsible for the original decrease in apoptosis, which, when knocked-down, led to an even more dramatic increase in cell death. Interestingly, cell viability decreased even more when p53 was knocked-down, likely due to the increase in apoptosis (Figure 5B). An examination of the cell cycle after knockdown of p53 (Figure 5C & 5D and Supplementary Figure 10B-10D) revealed that the G0/G1 and S phase populations decreased (from 73% to 61% and from 17% to 8%, respectively), whereas the G2/M and Sub G0 populations increased (rising from 7% to 18% and from 0 to 11%, respectively). This suggested that the cell cycle arrest had been rescued, but at the cost of dramatically higher levels of apoptosis. This also potentially explains why p53 was induced upon loss of GAS5. Given a potential link between GAS5 and p53 activities, qRT-PCR was performed, comparing the expression of p53 between IMR-32 cells and SK-N-AS cells (Supplementary Figure 11A). The results confirmed a dramatic difference in expression, with SK-N-AS cells expressing p53 at ~1% of that seen in IMR-32 cells. It is worth mentioning that LAN6 cells, which also responded to the loss of GAS5 by inducing cell cycle arrest, maintained expression of p53 at levels above that seen in IMR-32 cells (Supplementary Figure 11A). If the changes in apoptosis and cell viability due to the loss of GAS5 were dependent upon p53 activity, and SK-N-AS cells expressed p53 so poorly, this might explain why minimal phenotypes were seen from a reduction of GAS5 in this cell line. In order to determine if exogenous expression of p53 might rescue SK-N-AS cells, we cloned p53 from cDNA acquired from IMR-32 cells and then sub-cloned the gene into pcDNA6, forming the exogenous expression vector pcDNA6/p53. This vector was then co-transfected into SK-N-AS cells with GAS5 siRNA and compared to co-transfected cells with Negative Control siRNA and Vector only. qRT-PCR of p53 levels confirmed a 10-fold increase in expression vector–transfected cells compared to control vector cells (S11A), though this was still only a fraction of wild type gene expression in IMR-32 cells. Western blot analyses of the samples confirmed an increase in p53 protein expression, but only when in concert with GAS5 knock-down (Supplementary Figure 11B). An examination of the cell cycle (Figure 5E & 5F and Supplementary Figure 11C-11E) revealed that, whereas Negative Control cells with and without p53 showed minimal changes in G0/G1 (52% vs. 51%); the additional knockdown of GAS5 caused a dramatic increase in the G0/G1 population (up to 69%). Likewise, G2/M showed little difference with p53 expression alone (38% vs. 35%), whereas decreasing GAS5 reduced this population to half that of the controls (18%). Together, these data suggest that the SK-N-AS cells were undergoing cell cycle arrest. However, it is worth noting that no significant changes were seen in both Sub G0 and S phase, which distinguishes it from the results seen in IMR-32 cells. Regardless, these data support the hypothesis that reduction of GAS5 can induce p53 to activate cell cycle arrest in neuroblastoma cells. Furthermore, the knockdown of GAS5 in the SK-N-AS cell line did not induce cell cycle arrest due to a lack of p53.

### The loss of GAS5 in neuroblastoma cells modulates BRCA1 and GADD45A in order to induce cell cycle arrest

In order to better understand the mechanism(s) by which p53 was inducing cell cycle arrest in GAS5 knocked-down neuroblastoma cells, we decided to examine p53 pathway members utilizing a protein/phosphorylation ELISA array. This array (p53 Signaling – Phospho Antibody Microarray, Full Moon BioSystems) allowed us to simultaneously screen ~180 proteins both upstream and downstream of p53 activation as well as their phosphorylation-specific variants. IMR-32 cells were transfected with GAS5 or Negative Control siRNA for 48 hours and then harvested. A sample of these cell pellets were acquired and used to perform qRT-PCR on GAS5 and p53 to confirm both knockdown and upregulation, respectively (Supplementary Figure 12A & 12B). Western blotting was also performed to confirm an increase in p53 protein (Supplementary Figure 12C). The remaining cell samples were prepared for the ELISA array (Supplementary Figure 12D).

Analysis of the ELISA array revealed discrete phosphorylation events due to the loss of GAS5, confirming multiple occurrences of increased p53 phosphorylation (Supplementary Figure 13). Most surprising, however, was the fact that the top modulated candidate was not p53, but BRCA1 (BReast CAncer 1). Phosphorylation of BRCA1 occurred predominantly at ser1457, a site recognized as a target of ATM phosphorylation [22, 23]. Literature searches revealed that BRCA1 can not only physically interact with p53 [24], but can co-regulate p53-dependant gene expression [25]. In addition, co-activation of BRCA1 in an activated p53 background has been shown to predominantly shift cellular outcomes towards cell cycle arrest, as opposed to apoptosis [26]. Given that our results have overwhelmingly indicated this outcome as well, we returned to our RNA-Seq data and examined it for possible candidate genes downstream of p53 and/or BRCA1, which were transcriptionally activated by loss of GAS5 and could lead to induced cell cycle arrest. Since our search was so selective, it quickly revealed an obvious candidate: GADD45A (Growth Arrest and DNA Damage Inducible Alpha). GADD45A has been shown to be induced by both BRCA1 and p53 and is capable of activating both cell cycle arrest and apoptosis [26–29].

**Figure 6:**
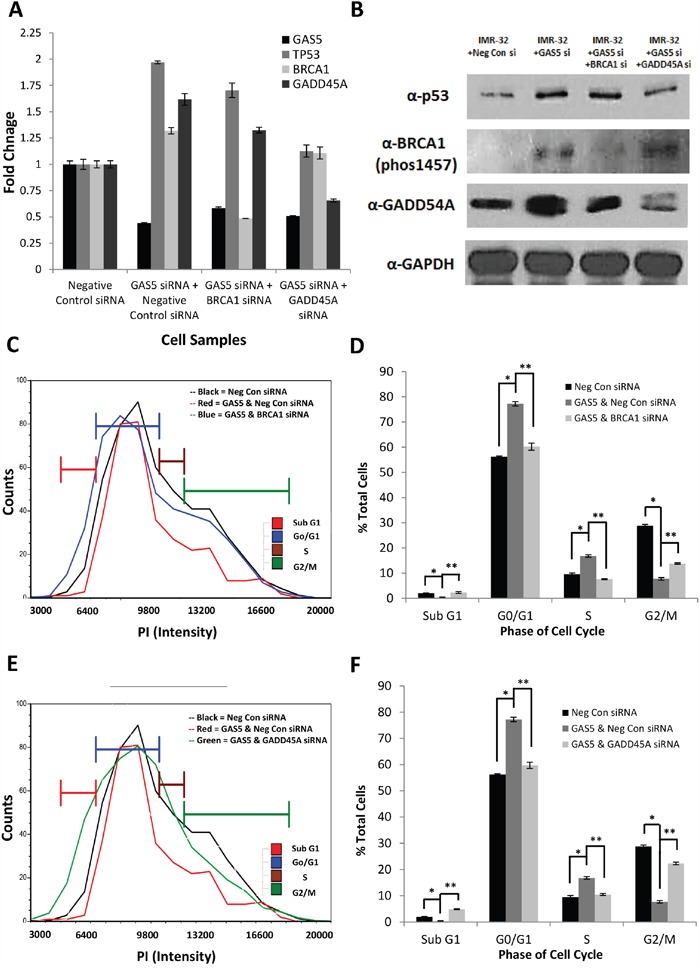
Effect of BRCA1 or GADD45A Co-Knockdown with GAS5 **A**. qRT-PCR of GAS5, p53, BRCA1, and GADD45A expression in IMR-32 cells after transfection with: Negative Control siRNA, GAS5 & Negative Control siRNA, GAS5 & BRCA1 siRNA, and GAS5 & GADD45A siRNA. Samples were normalized to the Ct value of GAPDH, with relative expression calculated by normalizing to the ΔΔCt of the Negative Control siRNA sample. Data were expressed as a means of +/- SD using three biological replicates. **B**. Western blot analysis of p53, BRCA1-phospho1457, and GADD45A in IMR-32 cells after transfection with: Negative Control siRNA, GAS5 & Negative Control siRNA, GAS5 & BRCA1 siRNA, and GAS5 & GADD45A siRNA. GAPDH was used as a load control. **C** and **D**. Measurement of cell cycle populations after knockdown with GAS5 and BRCA1 reveals limited rescue of cell cycle arrest with only a minimal increase in apoptosis. **E** and **F**. Measurement of cell cycle populations after knockdown with GAS5 and GADD45A reveals dramatic rescue of cell cycle arrest with greater increase in apoptosis. All experiments were performed in triplicate. * and ** p < 0.05, Student's t-test.

qRT-PCR and western blotting confirmed that knockdown of GAS5 led to increases in both the mRNA and protein content of p53, BRCA1, and GADD45A (Figure 6A & 6B & Supplementary Figure 14A-14C). Combinatorial knockdown of GAS5 and BRCA1 had no effect on p53 expression, but led to decreases in BRCA1 and GADD45A, suggesting that BRCA1 does have a regulatory effect on GADD45A. Analysis of the cell cycle in IMR-32 cells confirmed that additional knockdown of BRCA1 decreased G0/G1 and S phase populations (from 77% to 60% and from 16% to 7%, respectively), whereas the G2/M and Sub G0 populations increased (rising from 8% to 14% and from 0 to 2%, respectively) (Figure 6C & 6D, Supplementary Figure 15A-15C). This cell cycle rescue is similar to that seen with co-knockdown of p53, though the Sub G0 increase is far less profound (only 2% compared to 11%) and the G2/M recovery is somewhat diminished (14% instead of 18%), suggesting that the presence of p53 is suppressing apoptosis in a manner separate from BRCA1.

Interestingly, combinatorial knockdown of GAS5 and GADD45A did not significantly affect BRCA1, but did lead to some loss of p53 (though still higher than the negative control), in addition to GADD45A (Figure 6A & 6B & Supplementary Figure 14A-14C). This result is supported by previous reports that GADD45A can contribute to p53 stabilization [30]. Further, an analysis of the cell cycle confirmed that additional knockdown of GADD45A also decreased G0/G1 and S phase populations (from 77% to 60% and from 16% to 10%, respectively), whereas the G2/M and Sub G0 populations increased (rising from 8% to 22% and from 0 to 5%, respectively) (Figure 6E & 6F, Supplementary Figure 15A, 15B, & 15D). This rescue was more profound than that seen with BRCA1, with G0/G1 recovering above that seen with co-knockdown of either BRCA1 or p53 (22% compared to 14% or 18%, respectively), implying that GADD45A may be the primary factor to arrest the cell cycle in this instance.Oddly, the Sub G0 population increased compared to BRCA1, but was decreased compared to the p53 co-knockdowns (5% compared to 2% or 11%, respectively). This suggests, again, that the increased presence of p53 due to the loss of GAS5 is suppressing apoptosis and even a partial loss of p53 due to knockdown of GADD45A can affect its stability and thus, its suppression of apoptosis.

### Rescue of GAS5 knockdown cells with the FL but not the C2 splice variant stabilizes HDM2 leading to loss of p53 protein in neuroblastoma cells

**Figure 7:**
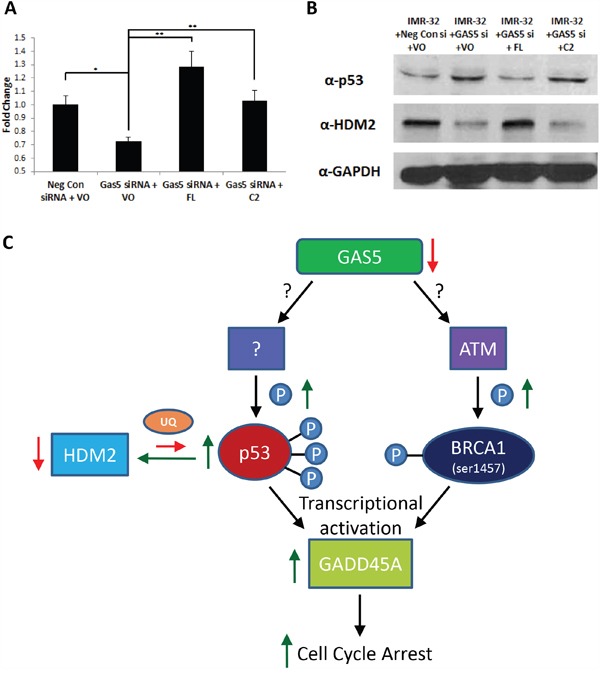
Complementation of FL splice variant in GAS5 knocked-down cells stabilizes HDM2 **A**. qRT-PCR of HDM2 (MDM2) expression in IMR-32 cells after transfection with: Negative Control siRNA & Vector only (VO), GAS5 siRNA + VO, GAS5 siRNA complemented with FL-expressing plasmid and GAS5 siRNA complemented with C2-expressing plasmid. Samples were normalized to the Ct value of GAPDH, with relative expression calculated by normalizing to the ΔΔCt of the Negative Control siRNA & Vector only sample. Data were expressed as a means of +/- SD using three biological replicates. **B**. Western blot analysis of p53 and HDM2 (MDM2) in IMR-32 cells after transfection with: Negative Control siRNA & Vector only (VO), GAS5 siRNA + VO, GAS5 siRNA complemented with FL-expressing plasmid, and GAS5 siRNA complemented with C2-expressing plasmid. GAPDH was used as a load control. * and ** p < 0.05, Student's t-test. **C**. Schematic illustration of the effects of GAS5 knockdown in neuroblastoma cells. Briefly, loss of GAS5 induces ser1457 phosphorylation of BRCA1, an ATM-specific phosphorylation site. Simultaneously, loss of GAS5 also induces multiple p53 phosphorylation events, leading to increased stability and a decrease in cellular HDM2 levels (likely due to auto- ubiquitination). Phospho-activated p53 and BRCA1 then induce increased transcription of GADD45A, leading to the induction cell cycle arrest.

Although p53 can be regulated via transcriptional mechanisms [31, 32], it is also highly regulated via post-translational modifications, such as ubiquitination [32], leading to the degradation of the p53 protein. The most well understood ubiquitin ligase known to target p53 is MDM2 (or HDM2, the human form of the protein) [32, 33]. An examination of RNA-Seq data from IMR-32 cells knocked-down for GAS5 revealed a possible coordination of expression between p53 and HDM2, revealing that the loss of GAS5 led to an increase in p53 mRNA and a subsequent decrease in HDM2 mRNA expression. Previous qRT-PCR had shown that p53 mRNA levels increased after loss of GAS5. qRT-PCR now confirmed that the loss of GAS5 also led to a decrease in HDM2 transcription (Figure 7A). In order to determine if protein levels corroborated transcript, western blot analysis was performed, revealing that p53 protein was in poor abundance under control conditions (Negative control siRNA and empty vector plasmid), but after knockdown with GAS5 siRNA, p53 protein increased considerably (Figure 7B & Supplementary Figure 16A & 16B). An examination of HDM2 protein content revealed that HDM2 was highly expressed under control conditions and severely lost after knockdown of GAS5, inversely correlating with p53 (Figure 7B). Complementation of the FL splice variant of GAS5 appeared to rescue GAS5 knockdown, increasing HDM2 transcript levels above even control conditions (Figure 7A), while simultaneously recovering lost HDM2 content and decreasing p53 protein content (Figure 7B). However, complementation with the C2 variant had little effect, recovering transcriptional losses of HDM2, but had no effect on either HDM2 or p53 protein expression, suggesting that transcriptional regulation of HDM2 by GAS5 cannot account for changes in protein content alone. An examination of p53 ubiquitination confirmed that complementation with the FL variant abrogated ubiquitination, whereas the C2 variant did not (Supplementary Figure 17). Together, these data indicate that the GAS5 FL variant can regulate HDM2 and p53, whereas the C2 variant cannot.

## DISCUSSION

Modern recognition of the function of lncRNAs in cell biology has introduced an entirely new tier of cell regulatory molecules, with estimates suggesting that as much as 80% of transcription involves products with little or no open reading frame [34, 35], though only for a fraction has a biological function been demonstrated [36]. Recent acknowledgement of lncRNA function has focused attention on disease etiology, and studies have since shown their involvement in neurological disease and oncogenesis [37–39]. Given recent discoveries of the presence of lncRNAs relevant to neuroblastoma [40, 41] and various reports of the significance of GAS5 in several cancer systems [8–10], we decided to examine if this lncRNA showed promise as a biomarker or possible therapeutic target in this cancer system.

An examination of MYCN-amplified and non-amplified neuroblastoma cell lines revealed robust expression of GAS5 in both, but with no initial correlation to MYCN expression. Characterization of GAS5 revealed several novel GAS5 splice variants present in both, but differentially expressed. The MYCN non-amplified cell line most highly expressed a full-length version of GAS5 (“FL”), whereas the MYCN-amplified cell line most highly expressed a novel variant containing a deletion in exon 7 (“C2”). Fluorescence *In situ* hybridization confirmed these variants were both nuclear in localization. Knockdown of GAS5 in MYCN-amplified cells led to losses in apoptosis and cell viability, as well as cell cycle changes indicating cell cycle arrest. Reintroduction of the individual variants after knockdown of total GAS5 revealed that the FL variant could rescue the changes in apoptosis, cell viability, and cell cycle arrest, whereas the C2 variant could not. The striking difference in both the expression and function of these variants suggests differing roles in cell regulatory pathways, and may explain why GAS5 has alternately been described as both a tumor suppressor and oncogene in various cancers.

An analysis by RNA-Seq after GAS5 knockdown revealed the top cellular networks modulated were associated with cell growth or proliferation, identifying p53 as a prime candidate, given a significant increase in its transcription and its known connections to these cellular phenotypes [18–21]. Combinatorial knockdown of GAS5 and p53 in MYCN-amplified cells revealed that the loss of p53 could rescue cell cycle arrest, decrease cell viability, and increase apoptosis, suggesting that the loss of GAS5 induces p53 to place the cell into cell cycle arrest in order to avoid inducing apoptosis. This was confirmed by reintroduction of exogenous p53 into SK-N-AS cells (which express p53 poorly), inducing cell cycle arrest after GAS5 knockdown. Further analysis confirmed increased p53 phosphorylation and a dramatic increase in BRCA1 phosphorylation (specifically at ser1457, previously identified as the target of ATM-dependent phospho-regulation of BRCA1) [22, 23], indicating that cellular responses due to the loss of GAS5 could be due to DNA damage or genomic instability. This hypothesis is further corroborated by the increased activation and stabilization of p53, whose functions are commonly associated with response to DNA damage [42–44]. Previous reports have shown that BRCA1 can physically interact with p53 and co-regulate its transcriptional targets, shifting cellular outcomes towards cell cycle arrest [24–26]. Co-knockdown of BRCA1 with GAS5 also led to a partial rescue of arrest, with a minimal effect on apoptosis, suggesting that the role of BRCA1 in this instance is not to suppress apoptosis, but to assist in inducing arrest. Downstream analysis of p53 and BRCA1 target genes identified GADD45A as a possible mechanistic candidate since it is capable of activating both cell cycle arrest and apoptosis [26–29]. Knockdown of GAS5 significantly up-regulated its expression, which was partially abrogated by the loss of BRCA1. Co-knockdown of GADD45A and GAS5 led to a significant rescue of arrest, with some commensurate increase in apoptosis, suggesting that GADD45A primarily regulates arrest in this instance.

Further investigation of p53 regulation revealed an inverse correlation with MDM2 (HDM2), a ubiquitin ligase known as a primary regulator of p53 [32, 33]. Loss of GAS5 led to increases in both transcript and protein content of p53, with likewise decreases in HDM2. Given that a primary mechanism of regulation by HDM2 involves ubiquitination of p53 [33], our data support that p53 protein accumulation is due to a lack of ubiquitination by HDM2, possibly due to increased p53 phosphorylation. A schematic representation of the cellular response pathway described in this manuscript upon loss of GAS5 is illustrated in Figure 7C. Complementation with the GAS5 FL variant rescued the expression changes in p53 and HDM2, whereas the C2 variant could not, supporting a possible mechanism of rescue from cell cycle arrest, with the FL variant biasing the cell toward loss of p53 and lack of check-point control in cell proliferation. It is possible that a transition from C2 to FL variant expression may drive a neuroblastoma toward more aggressive behavior by assisting the cell in ignoring genomic instability caused by the transformation process and inhibiting the tumor suppressor functions of p53 and BRCA1. Traditionally, p53 is not considered clinically relevant to the treatment paradigm of neuroblastoma. In the 2011 paper by Van Maerken, only ~26% of neuroblastoma cell lines examined contained mutations in the p53 gene [45]. These data support the hypothesis that defects in p53 activation may be more prevalent than the incidence of p53 mutations which are commonly associated with its loss of function. In this incidence, the FL splice variant of GAS5 can release cell cycle arrest after loss of total GAS5 in a p53-dependent manner. The ability of lncRNAs to modulate p53 is not unheard of: NEAT1 has previously been shown to promote signaling that attenuates activation of p53 in human cancer cells [46] and N1LR has been shown to enhance the cell cycle and proliferation of neuronal cells via inhibition of p53 phosphorylation [47]. Previous studies of GAS5 have indicated differential regulatory control of both apoptosis and the cell cycle in various human cancers [13–17]. However, given evidence of over two dozen splice variants for GAS5, it is likely that discrete control over these mechanisms may be variant and cell type specific, suggesting that the inconsistent regulatory patterns seen between cancers may be a result of differential variant expression, and thus, their differential control over these pathways. Thus, it is our belief that the abundant patterns of splice variants for GAS5 are not simply a transcriptional anomaly, but an action of directed splicing whose purpose is to offer a broader variation of control over various cellular activities, which, based upon our own data, include cell cycle regulation and arrest. What may be witnessed in other cancers may include a different profile of variants, which are typically quantified as a group, but when isolated and analyzed separately, may offer entirely new insights into unique splice variant-specific functions. Given our own results, we would suggest more attention be given to the variant profiles present and the phenotypes they produce.

Our results are the first to confirm that splice variants of a lncRNA differentially regulate cell-cycle check-point control in human neuroblastoma. More importantly, these studies offer a new option for diagnostic and therapeutic targets in the difficult to treat MYCN-amplified background. Future studies will require more attention given to the direct targets of GAS5 and the mechanism by which the FL variant is capable of regulating cell cycle control as well as a patient sample study to elucidate the diagnostic and prognostic potential of the GAS5 splice variants in neuroblastoma treatment.

## MATERIALS AND METHODS

### Cell lines

IMR-32 cells were cultured in Minimum Essential Medium (MEM) Alpha + GlutaMAX™ [Gibco Life Sciences] supplemented with 10% fetal bovine serum [FBS]. SK-N-AS cells were cultured in Dulbecco's modified Eagle's medium (DMEM) [Gibco Life Sciences] supplemented with 10% FBS and 1% non-essential amino acids (NEAA). IMR-32 and SK-N-AS cells were purchased from ATCC. Further, we obtained an additional 10 cell lines from Children's Oncology Group, which were cultured as follows: CHLA-15, CHLA-42, CHLA-90, CHLA-122, CHLA-171 and NB-EBc1 cells were cultured in HyClone Iscove's Modified Dulbecco's Medium (IMDM) [GE Healthcare Life Sciences] supplemented with 20% FBS and 1X ITS (5 μg/mL insulin, 5 μg/mL transferrin, 5 ng/mL selenous acid). LA-N-6, SK-N-Be(1), SK-N-FI and SMS-KAN cells were cultured in HyClone RPMI-1640 [GE Healthcare Life Sciences] supplemented with 10% FBS. All cells were incubated and maintained at 37°C and 5% CO_2_. GI-M-EN, KCNA, NPE and SK-N-SH RNA were graciously provided to us by Vid Mlakar and Fabienne Gumy-Pause (Cansearch, Faculty of Medicine, University of Geneva).

### cDNA synthesis and real time qRT-PCR

For cell lines, total RNA (500 ng) was acquired using a miRNeasy Mini Kit (Qiagen) and reverse transcribed using a High Capacity cDNA Reverse Transcription kit (Applied Biosystems, Foster City, CA) following the manufacturer's instructions.

Quantitative PCR was performed using SYBR Green mRNA assays on a 7500 Real-Time PCR System (Applied Biosystems/Life Technologies), according to the manufacturer's instructions. SDS 2.3 software (Applied Biosystems) was used for comparative Ct analysis, with GAPDH used as the endogenous control. Primers for SYBR qPCR were as follows: GAS5 qPCR For, gaccacaacaagcaagcatgcagc; GAS5 qPCR Rev, gcttcaaaggccactgcactctagc; GAS5 FL qPCR For, ggacatgaagacagttcctgtcatacc; GAS5 FL qPCR Rev, gactcagaattcatgattgaagaaatgc; GAS5 C2 qPCR For, catggatgacttgcttgggtatggag; GAS5 C2 qPCR Rev, aggataacaggtctgcctgcatttc; GAS5 C3 qPCR For, ggacatgaagacagttcctgtcatacc; GAS5 C3 qPCR Rev, tgtggtcattaaaaaccctttgcttc; GAS5 C4 qPCR For, catggatgacttgcttgggtatggag; GAS5 C4 qPCR Rev, tggtcattaaaaaccctgcatttcttc; BRCA1 qPCR For, gtgcagccagatgcctggacag; BRCA1 qPCR Rev, cagctcctggcactggtagagtgc; GADD45A qPCR For, cctgcactgcgtgctggtgac; GADD45A qPCR Rev, ccatgtagcgactttcccggc; HDM2 qPCR For, gtagcagtgaatctacagggacgcc; HDM2 qPCR Rev, cctgatccaaccaatcacctgaatg; TP53 qPCR For, ctcaaggatgcccaggctggg; TP53 qPCR Rev, tatggcgggaggtagactgaccc.

### siRNA knockdown in cells

IMR-32 and SK-N-AS cells were trypsinized and reverse transfected using Lipofectamine® RNAiMax (Life Technologies) and 50 μM of the following siRNAs (Life Technologies): GAS5 Silencer® Select siRNA (cat #: n272331), TP53 Silencer® Select siRNA (cat #: s607), BRCA1 Silencer® Select siRNA (cat #: s459), GADD45A Silencer® Select siRNA (cat #: s225791), and Silencer® Select negative control #1 (cat #: 4390843). Cells were seeded into 6-well plates at a density of 2.5 × 10^5^ cells per well. Cells were incubated for 24 or 48 hours at 37°C in a CO_2_ incubator. Once the transfection period was complete, cells were harvested and counted for use in cell biology assays. The efficiency of gene knockdown was assessed by quantitative real-time PCR (qRT-PCR).

### Plasmid construction & transfection

Total RNA was isolated from IMR-32 cells using a miRNeasy Mini Kit (Qiagen) and reversed transcribed using M-MLV reverse transcriptase. The cDNA was then used as a template for PCR amplification using GoTaq (Promega) with primers designed as follows: GAS5 cloning For (BamHI) – tggatcctttcgaggtaggagtcgactcctgtg and GAS5 cloning Rev (XhoI) – actcgagtggattgcaaaaatttattaaaattggag or TP53 clone For (Hind III) – ttttttaagcttgccaccatggaggagccgcagtcagatc and TP53 clone Rev (Xho I) - aaaaaactcgagtcagtctgagtcaggcccttctgtc. PCR products were gel purified (QIAquick Gel Extraction kit, Qiagen), TOPO-cloned into pCR4-TOPO (Life Technologies), transformed into Top10 Chem comp cells and plated on LB Amp plates (100 ug/mL). Colonies were grown in LB Amp (100 ug/mL) O/N at 37°C, then plasmids were harvested by miniprep (QIAprep spin miniprep kit, Qiagen). Clones were sequenced (Retrogen) and analyzed using VectorNTi and AlignX (Life Technologies). The GAS5 splice variants were then sub-cloned into pcDNA6/V5-HisA by restriction digestion using BamHI and XhoI. The TP53 ORF was sub-cloned into pcDNA6/V5-HisA by restriction digestion using HindIII and XhoI. All clones were ligated using T4 Ligase (NEB, Inc.), confirmed by restriction digestion and quantified for transfection. Both p53 and the FL GAS5 splice variant were confirmed as containing no mutations compared to known sequences as listed by Ensembl.

IMR-32 or SK-N-AS cells were seeded into single wells of a 6-well plate at a density of 2.5 × 10^5^ cells per well and transfected with 1 μg of either pcDNA6/V5-HisA (Vector Control), pcDNA6/C2 (C2 variant) or pcDNA6/FL (FL variant), or pcDNA6/p53 using FuGENE® 6 (Promega).

### Apoptosis assay

IMR-32 and SK-N-AS cells were reverse transfected and counted as described above. Cells were then seeded into 96-well plates at a density of 1 × 10^4^ and 1.5 × 10^4^ cells per well. Caspase activity for GAS5 knockdown was then measured using the Caspase-Glo® 3/7 Assay kit (Promega) according to the manufacturer's recommendations, and samples were read on a GloMax luminometer (Promega) in triplicate.

### Cell proliferation assay

Cells were reverse transfected and seeded as described above. CellTiter 96® AQueous One Solution Cell Proliferation (3-(4,5-dimethylthiazol-2-yl)-5-(3-carboxymethoxyphenyl)-2-(4-sulfophenyl)-2H-tetrazolium, inner salt; MTS) assay was purchased from Promega (Madison, WI) and used according to the manufacturer's instructions. Samples were read on a SpectraMax M5 (Molecular Devices Corp.) system at a wavelength of 490nm using SoftMax Pro (version 6.2.1) software.

### Cell cycle assay

IMR-32 and SK-N-AS cells were reverse transfected as described above. Cell cycle analysis was performed using the Cellometer™ Propidium Iodide (PI) Cell Cycle Kit (Nexcelom Bioscience), according to the manufacturer's instructions, on a Cellometer Vision instrument with Cellometer Vision CBA software (version 2.1.4.2) and De Novo FCS Express 4 software for analysis.

### RNA-FISH analysis

5’-biotin labeled probes for human lncRNA GAS5 FL (ggtatgacaggaactgtcttcatgtcc) and lncRNA GAS5 C2 (cgactctccatacccaagcaagtc) were used for RNA–FISH. *In situ* hybridization was performed on a Ventana machine using RiboMap in situ hybridization kit (Ventana Medical Systems, Inc.). The cell suspension was diluted to 10,000 cells/100 μL and plated on autoclaved glass slides. The next day, slides were washed in PBS and fixed in 4% paraformaldehyde and 5% acetic acid. The acid treatment consisted of hydrochloride-based RiboClear reagent (Ventana Medical Systems) for 10 min at 37°C, followed by treatment with protease 3 reagent. After an initial denaturing prehybridization step for 4 min at 80°C, the cells were hybridized with the antisense streptavidin-FITC conjugate (40 nmol/L) using RiboHybe hybridization buffer (Ventana Medical Systems) for 2 hours (at 59°C for the C2 probe and at 62°C for the FL probe). Next, the slides went through a low-stringency wash (0.1× SSC; Ventana Medical Systems) for 4 min at 60°C, and then 2 further washing steps (1× SSC) for 4 min at 60°C. Slides were fixed in RiboFix and counterstained with 4′-6′diamidino-2-phenylindole (DAPI), in Prolong Gold® antifade reagent. The images were captured on a Nikon A1R VAAS laser point- and resonant-scanning confocal microscope outfitted with a single photon Ar-ion laser at 60× with 4× zoom.

### Western blot analysis

Total lysates of 5×10^5^ cells/each cell treatment were boiled under denaturing conditions and proteins separated on 6% Tris-Glycine denaturing polyacrylamide gels by electrophoresis. Proteins transferred to nitrocellulose membranes were probed with the following primary antibodies: anti-p53 (Cell Signaling Technology, Inc.) at 1/200, anti-HDM2 (MDM2) (EMD Millipore) at 1/500, anti-BRCA1 (phosphor-ser1457) (Biorbyt, Cat # orb125493) at 1/1000, anti-GADD45A (Cell Signaling Technology, Cat # 4632S) at 1/500, and anti-GAPDH (Santa Cruz, FL-335) at 1/2000 according to standard methods. Blots were probed with horseradish peroxidase-conjugated secondary antibodies and visualized with ECL chemiluminescence (Pierce) or Alexa 680-conjugated secondary antibodies (Molecular Probes) and visualized on the Licor Odyssey (Licor).

### Next-generation RNA sequencing

Total RNA was isolated using Trizol reagent (Ambion, Foster City, CA) and quantified with a ND-1000 spectrophotometer (Thermo Scientific, Wilmington, DE). The quality of Total RNA was assessed by the Agilent Bioanalyzer Nano chip (Agilent Technologies). RNA-Seq library was constructed using the Truseq Stranded Total RNA library preparation instruction from Illumina using 1ug of Total RNA. Shortly, the total RNA is Ribo-depleted using Ribo-Zero Gold kit to remove rRNA from total RNA and then fragmented into small pieces using divalent cations under elevated temperature. Following fragmentation, the first strand cDNA were synthesized using random primers and followed by second strand synthesis using DNA Polymerase I. The cDNA is then ligated with index adapters for each sample followed by purification and then enriched with PCR to create the final library. The quality and quantity of the libraries were detected by Agilent Bioanalyzer and Kapa Biosystems qPCR. Multiplexed libraries are pooled and single-end 50-bp sequencing was performed on one flow-cell of an Illumina Hiseq 1500. The raw reads (fastq files) from Illumina HiSeq1500 were aligned to the reference genome using TopHat version 1.4.1 ( http://www.ncbi.nlm.nih.gov/pubmed/19289445) with RefSeq annotations and the “—no-novel-juncs” option. Ambiguous reads that mapped to more than one region in the genome and reads with MAPQ score less than 10 were removed. The UCSC human genome version 19 (hg19) and corresponding RefSeq annotations were used for reference and mRNA transcript quantification. The bed coordinates of lncRNAs from the Dinger Lab were applied for lncRNA transcript quantification. Transcript quantification was performed using Partek Genomics Suite (version 6.4, Partek Inc, St. Louis MI), and the raw read counts and normalized read counts (RPKM: reads per kilobase per million mapped reads, ( https://www.ncbi.nlm.nih.gov/geo/query/acc.cgi?acc=GSE90523). were obtained. The raw count information for all the mRNA and lncRNA transcripts was first filtered, with transcripts that did not produce read counts in all the samples filtered from further analyses. The remaining transcripts were analyzed using the BioConductor DESeq package ( http://www.ncbi.nlm.nih.gov/pubmed/20979621) to detect differential expression between GAS5 knockdowns and negative control cells. Transcripts detected in at least one sample (RPKM>1), fold change over 1.5, and with a p-value less than 0.05 were considered significantly differentially expressed. These experiments were performed with two replicates for all samples.

### P53 pathway phospho antibody microarray

IMR-32 cells were seeded and transfected in 6 well plates with GAS5 Silencer® Select siRNA n272331 or Silencer® Select negative control #1 (as described above; siRNA Knockdown, using 12 wells per sample). After 48 hours, the cell samples were lifted in PBS, then counted, with 1.5 × 10^6^ and 10^6^ cells aliquoted separately. The aliquots were spun down and the PBS removed, then frozen at -80°C for future use in the array or for western blot analysis. The remaining cells were prepared for total RNA using a miRNeasy Mini Kit (Qiagen). Following validation of GAS5 knockdown and p53 increased expression by qRT-PCR, western blot analysis was performed (using the 10^6^ cell pellets) to confirm gain of p53 and loss of HDM2. The remaining cell pellet (containing 1.5 × 10^6^ cells) was prepared according to manufacturer's protocols (Antibody Array Assay Kit, Cat. No. KAS02, Full Moon BioSystems) and coupled to an ELISA-based array platform (p53 Signaling – Phospho Antibody Microarray, Cat. No. PFT196, Full Moon BioSystems). Array images were acquired using a SureScan Microarray Scanner (Agilent Technologies) at 10-micron resolution. Image analysis was performed using a GenePix Array list (GAL) file by Full Moon BioSystems and array results were delivered as an Excel file.

### Ubiquitination detection assay

IMR-32 cells were seeded and transfected in 6 well plates (see above *siRNA Knockdown in cells*) with GAS5 Silencer® Select siRNA or Silencer® Select Negative Control #1, as well as pcDNA6/V5-HisA, pcDNA6/FL, or pcDNA6/C2. Forty-eight hours after transfection, samples were harvested and ubiquitinated proteins were detected and visualized as performed in Choo *et al*. [48]

## SUPPLEMENTARY MATERIALS FIGURES


