# Supplementary material for: The long non-coding RNA GAS5 differentially regulates cell cycle arrest and apoptosis through activation of BRCA1 and p53 in human neuroblastoma
Source: Oncotarget. 2016 Dec 27;8(4):6589–607. doi: 10.18632/oncotarget.14244 (PMC5351655; doi:10.18632/oncotarget.14244)
Supplement: Supplementary file 1 [file oncotarget-08-6589-s001.pdf]

The long non-coding RNA GAS5 differentially regulates cell cycle arrest and apoptosis through activation of BRCA1 and p53 in human neuroblastoma

SUPPLEMENTARY FIGURES

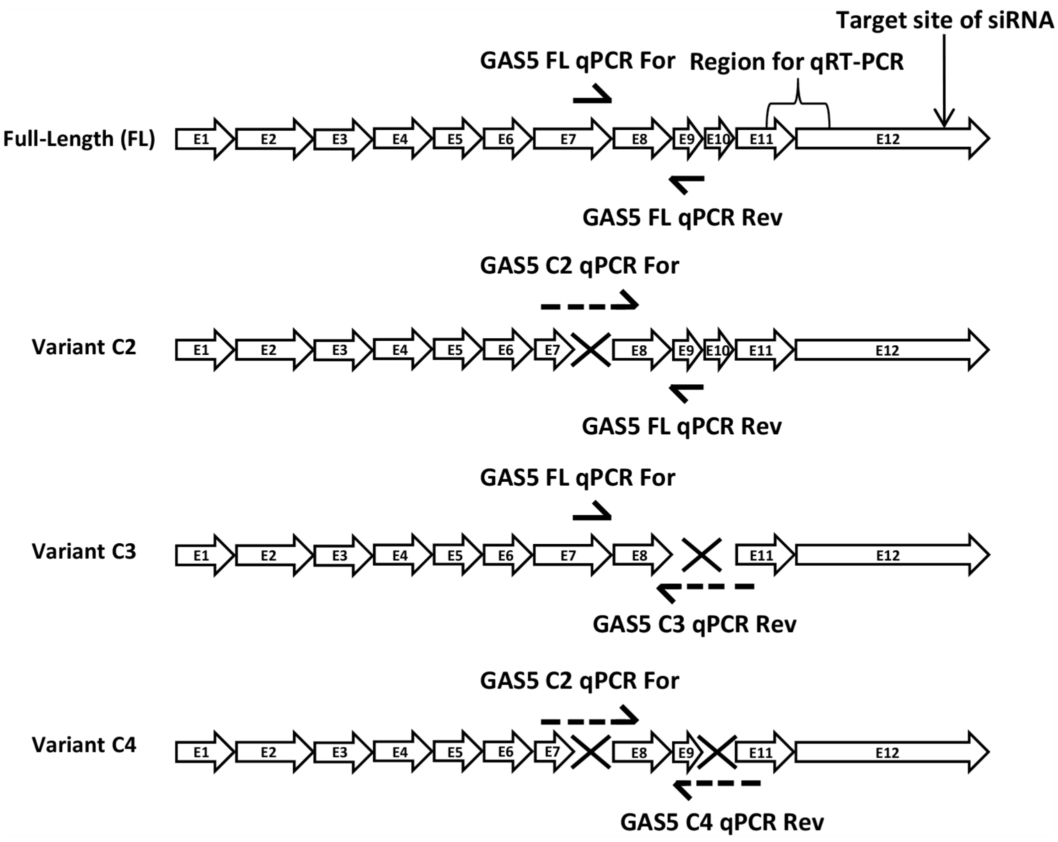

Supplementary Figure 1: Schematic illustration of the GAS5 "Full-Length" and three novel splice variants discovered in human neuroblastoma cells and the locations of the qRT-PCR primer sets used to distinguish them. The FL ("Full-Length") splice variant was quantified using primers that annealed to exons 7 and 9. The C2 variant used a forward primer which crossed from the front end of exon 7 through to exon 8. This allowed for primer annealing only if the second half of exon 7 was not present. The C3 variant used a reverse primer which crossed from the front end of exon 11 backward to second half of exon 8. This allowed for primer annealing only if exons 9 and 10 were not present. And finally, the C4 variant used the previously used C2 forward primer and a reverse primer which crossed from the front end of exon 11 backward to through exon 9. This allowed for primer annealing only if exon 10 was not present.

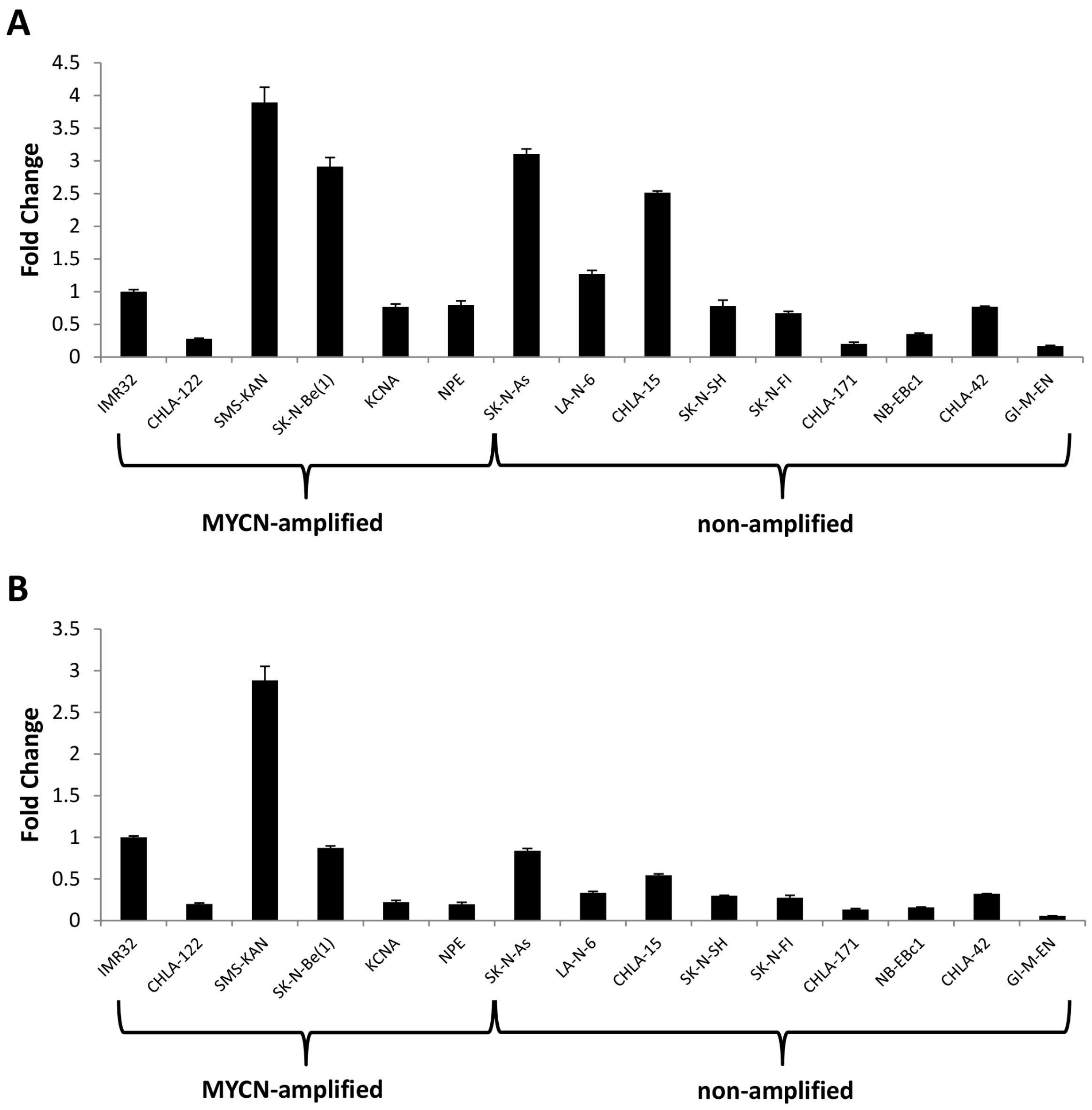

Supplementary Figure 2: Expression of the FL ("Full-Length") and C2 ("Clone 2") splice variants in human neuroblastoma. A. FL and **B.** C2 expression in neuroblastoma cell lines as measured by qRT-PCR. FL and C2 expression is confirmed in the MYCN-amplified cell lines IMR-32, CHLA-122, SMS-KAN, SK-N-Be(1), KCNA, and NPE cell lines as well as in the non-MYCN-amplified cell lines SK-N-AS, LA-N-6, CHLA-15, SK-N-FI, CHLA-171, NB-EBc1, CHLA-42, and GI-M-EN. The Ct value of each sample was normalized to the Ct value of GAPDH, and the relative expression was calculated by normalizing to IMR-32 cells by calculating the ΔΔCt method. Data are expressed as means +/- SD from three biological replicates for each sample.

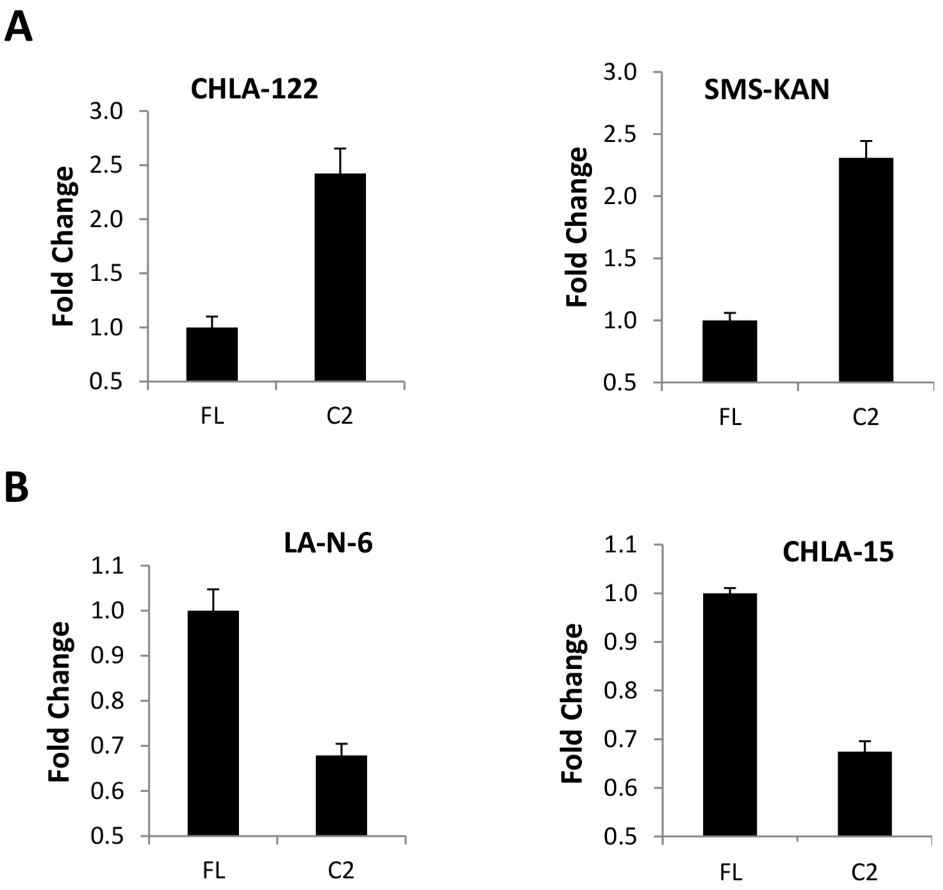

Supplementary Figure 3: Further examination of GAS5 splice variant expression in MycN-amplified vs. non-amplified neuroblastoma cells. A. Comparison of FL vs. C2 GAS5 splice variant expression as measured in the MYCN-amplified cell lines CHLA-122 and SMS-KAN by qRT-PCR. **B.** Comparison of FL vs. C2 GAS5 splice variant expression as measured in the non-amplified cell lines LA-N-6 and CHLA-15 by qRT-PCR. Cell samples were normalized to the Ct value of GAPDH and relative expression was calculated by normalizing to the ΔΔCt of the FL variant. Data are expressed as means +/- SD using three biological replicates for each.

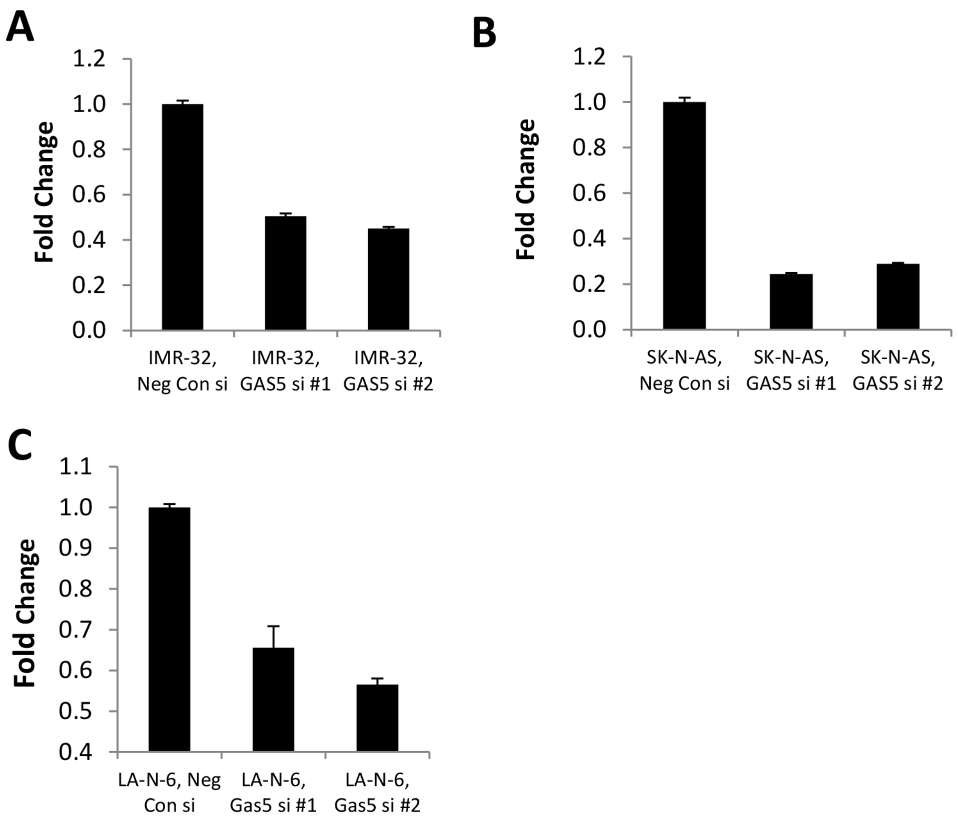

Supplementary Figure 4: Examination of the knock-down efficiency of GAS5 siRNAs in neuroblastoma cells as measured by qRT-PCR. A. Compared to Negative Control siRNA, efficiency of knock-down with two separate GAS5 siRNAs was confirmed at ~51% and 55% in IMR-32 cells after 24 hrs. **B.** Compared to Negative Control siRNA, efficiency of knock-down with two separate GAS5 siRNAs was confirmed at ~76% and 71% in SK-N-AS cells after 24 hrs. **C.** Compared to Negative Control siRNA, efficiency of knock-down with two separate GAS5 siRNAs was confirmed at ~34% and 43% in LA-N-6 cells after 24 hrs. Each sample was normalized to the Ct value of GAPDH. The relative expression was then calculated by normalizing to the ΔΔCt of the cell sample + Negative Control siRNA. All data are expressed as means +/- SD using three biological replicates for each.

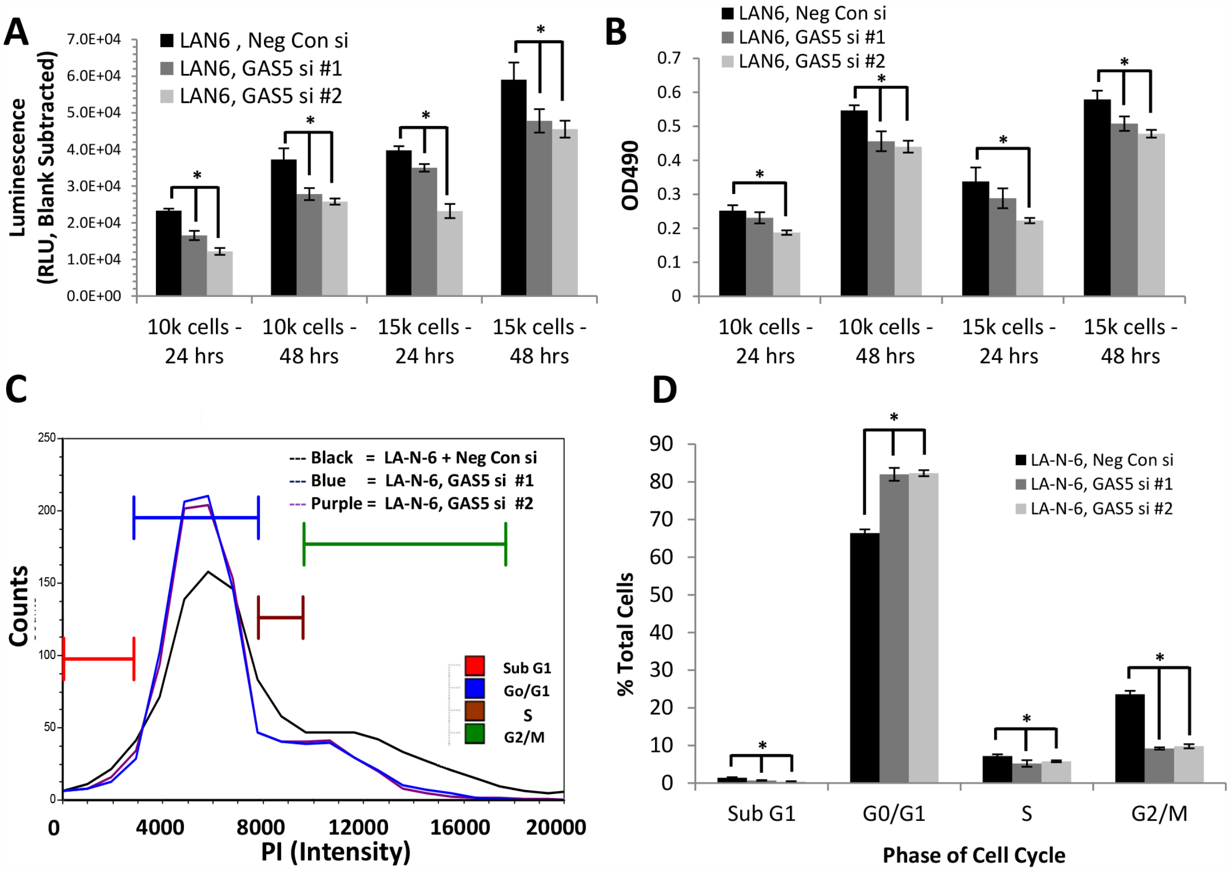

Supplementary Figure 5: Effect of GAS5 knockdown on LA-N-6 neuroblastoma cells. A. Loss of GAS5 in LA-N-6 cells decreases apoptosis rate at both 24 and 48 hr time points. **B.** Loss of GAS5 in LAN6 cells decreases cell proliferation rate at both 24 and 48 hr time points. **C** and **D.** Knockdown of GAS5 in LA-N-6 cells increases the content of G0/G1 cells and decreases the content of G2/M phase cells, inducing cell cycle arrest, as measured by propidium iodide staining. All experiments were performed in triplicate. * $P < 0.05$, Student's t-test.

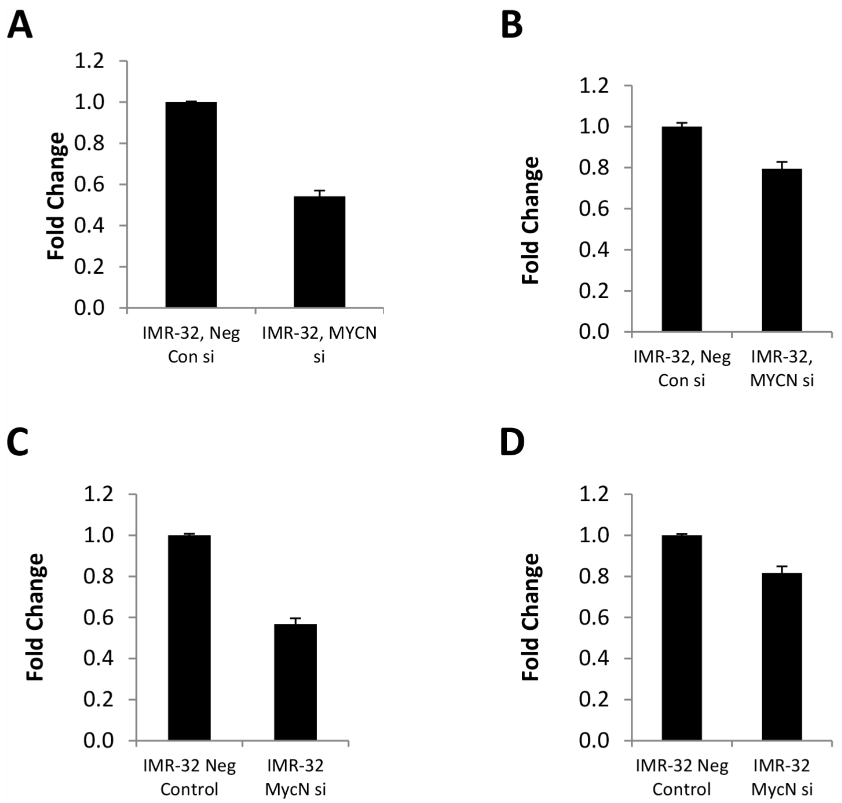

Supplementary Figure 6: Effect of MYCN knock-down on total GAS5 and GAS5 splice variant expression in neuroblastoma cells as measured by qRT-PCR. A. Compared to Negative Control siRNA, the efficiency of knock-down by MYCN siRNA was confirmed at ~54% in IMR-32 cells after 48 hrs. **B.** Due to knock-down of MYCN, total GAS5 gene expression was measured at ~79% compared to Negative Control siRNA after 48 hrs. **C.** Due to knock-down of MYCN, GAS5 FL variant gene expression was measured at ~57% compared to Negative Control siRNA after 48 hrs. **D.** Due to knock-down of MYCN, GAS5 C2 variant gene expression was measured at ~82% compared to Negative Control siRNA after 48 hrs. Each sample was normalized to the Ct value of GAPDH. The relative expression was then calculated by normalizing to the ΔΔCt of the cell sample + Negative Control siRNA. All data are expressed as means +/- SD using three biological replicates for each.

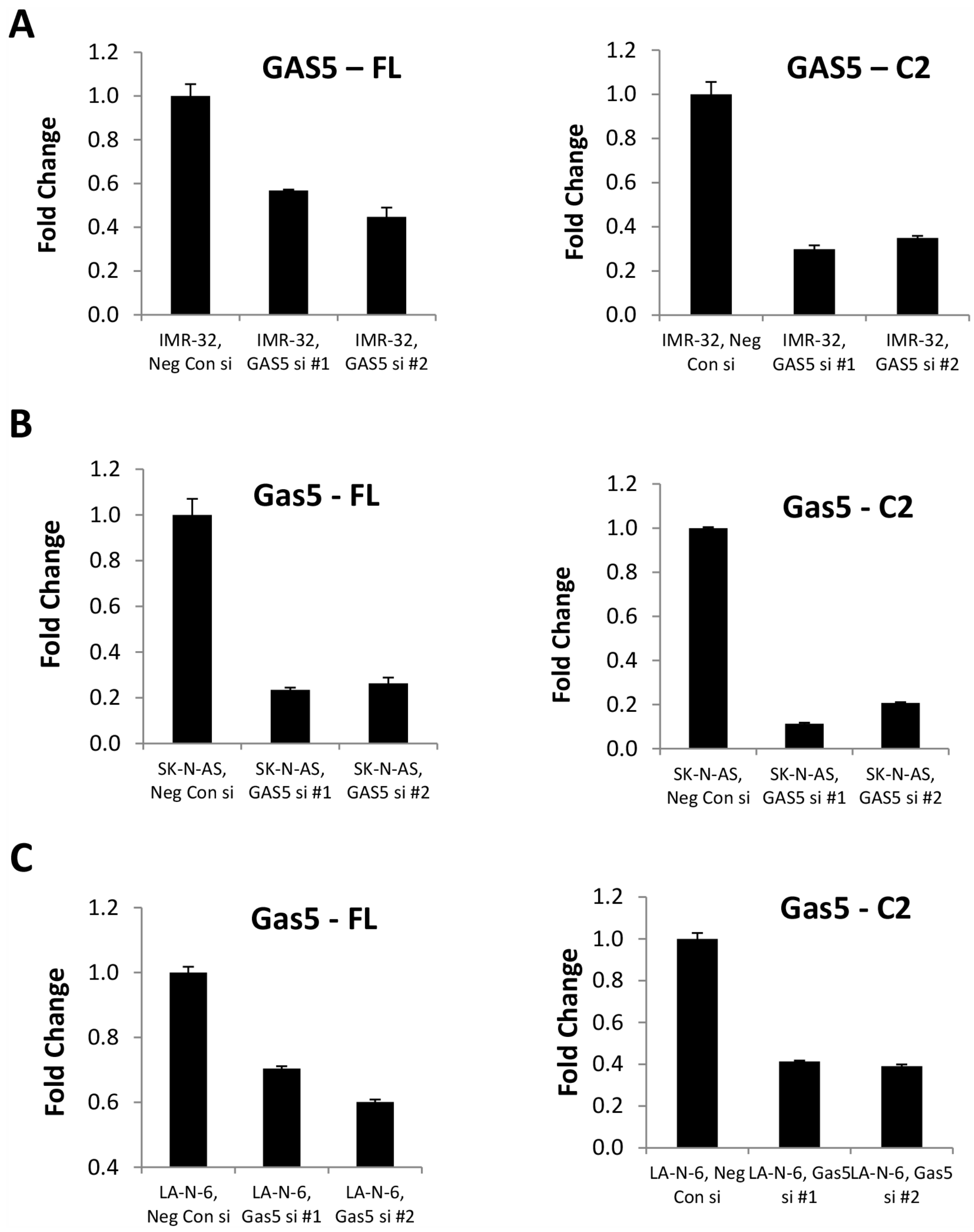

Supplementary Figure 7: Confirmation of GAS5 splice variant knock-down by GAS5 siRNA. A. Examination of the knock-down efficiency of two separate GAS5 siRNAs compared to Negative Control siRNA on both FL and C2 GAS5 splice variants in IMR-32 cells by qRT-PCR. **B.** Examination of the knock-down efficiency of two separate GAS5 siRNAs compared to Negative Control siRNA on both FL and C2 GAS5 splice variants in SK-N-AS cells by qRT-PCR. **C.** Examination of the knock-down efficiency of two separate GAS5 siRNAs compared to Negative Control siRNA on both FL and C2 GAS5 splice variants in LA-N-6 cells by qRT-PCR. Both cell line samples were normalized to the Ct value of GAPDH, with relative expression calculated by normalizing to the ΔΔCt of the Negative Control siRNA of the appropriate cell line. Data were then expressed as a means of +/- SD using three biological replicates.

A

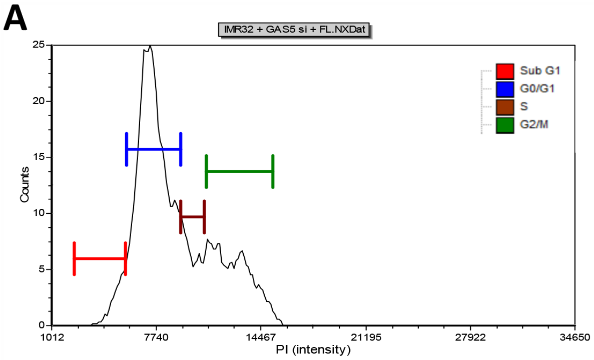

Cell Population	% of Gated Cells	CV
Total	100 %	
Sub G1	4.3 %	7.7 %
G0/G1	59.7 %	11.2 %
S	10.7 %	4.8 %
G2/M	23.3 %	8.8 %

B

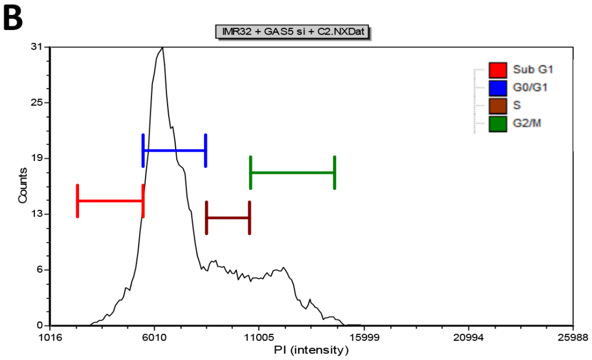

Cell Population	% of Gated Cells	CV
Total	100%	
Sub G1	6.0%	11.4 %
G0/G1	67.9%	10.9 %
S	13.7%	6.2 %
G2/M	11.2%	7.6 %

Supplementary Figure 8: Effect of FL or C2 splice variant complementation on GAS5 knock-down of IMR-32 cells as measured by the Cell Cycle. A. Individual trace of GAS5 FL splice variant complementation in IMR-32 cells. Labeling by propidium iodine staining indicated cell populations of 4.3% in Sub G1, 59.7% in G0/G1, 10.7% in S phase, and 23.3% in G2/M. **B.** Individual trace of GAS5 C2 splice variant complementation in IMR-32 cells. Labeling by propidium iodine staining indicated cell populations of 6.0% in Sub G1, 67.9% in G0/G1, 13.7% in S phase, and 11.2% in G2/M. All experiments were performed in triplicate.

A

Top Networks

ID	Associated Network Functions	Score
1	Hereditary Disorder, Metabolic Disease, Neurological Disease	49
2	RNA Post-Transcriptional Modification, RNA Damage and Repair, Developmental Disorder	46
3	Gene Expression, Protein Synthesis, Post-Translational Modification	41
4	Carbohydrate Metabolism, Molecular Transport, Cell Death and Survival	39
5	Cellular Assembly and Organization, Cellular Function and Maintenance, Cell-To-Cell Signaling and Interaction	32

Top Diseases and Bio Functions

Diseases and Disorders

Name	p-value	#Molecules
Cancer	1.88E-03 - 6.03E-23	981
Organismal Injury and Abnormalities	1.88E-03 - 6.03E-23	988
Gastrointestinal Disease	1.39E-03 - 2.11E-20	733
Reproductive System Disease	4.92E-04 - 2.48E-18	505
Neurological Disease	1.93E-03 - 1.73E-16	322

Name	p-value	#Molecules
Cellular Assembly and Organization	1.81E-03 - 2.67E-16	259
Cellular Function and Maintenance	1.39E-03 - 2.67E-16	297
Cellular Development	1.91E-03 - 4.26E-14	387
Cell Morphology	1.46E-03 - 4.38E-14	288
Cellular Growth and Proliferation	1.91E-03 - 1.27E-13	391

B

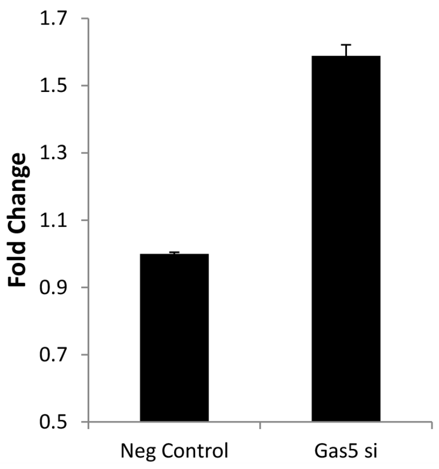

Supplementary Figure 9: Analysis of Global Network changes as measured by RNA-Seq in IMR-32 cells knocked-down for GAS5. A. Ingenuity Pathway Analysis (IPA) analysis of Top Networks modulated by the loss of GAS5, followed by Top Diseases and Bio Functions. The results indicate that the loss of GAS5 most highly correlated with changes associated with hereditary, metabolic, or neurological diseases most likely cause by Organismal Injury or Cancer, specifically involving Cell Growth, Proliferation, or Development. **B.** Validation of the induction of p53 mRNA seen in RNA-Seq data upon the loss of GAS5 in IMR-32 cells by qRT-PCR. Samples were normalized to the Ct value of GAPDH, with relative expression calculated by normalizing to the ΔΔCt of the Negative Control siRNA sample. Data were expressed as a means of +/- SD using three biological replicates.

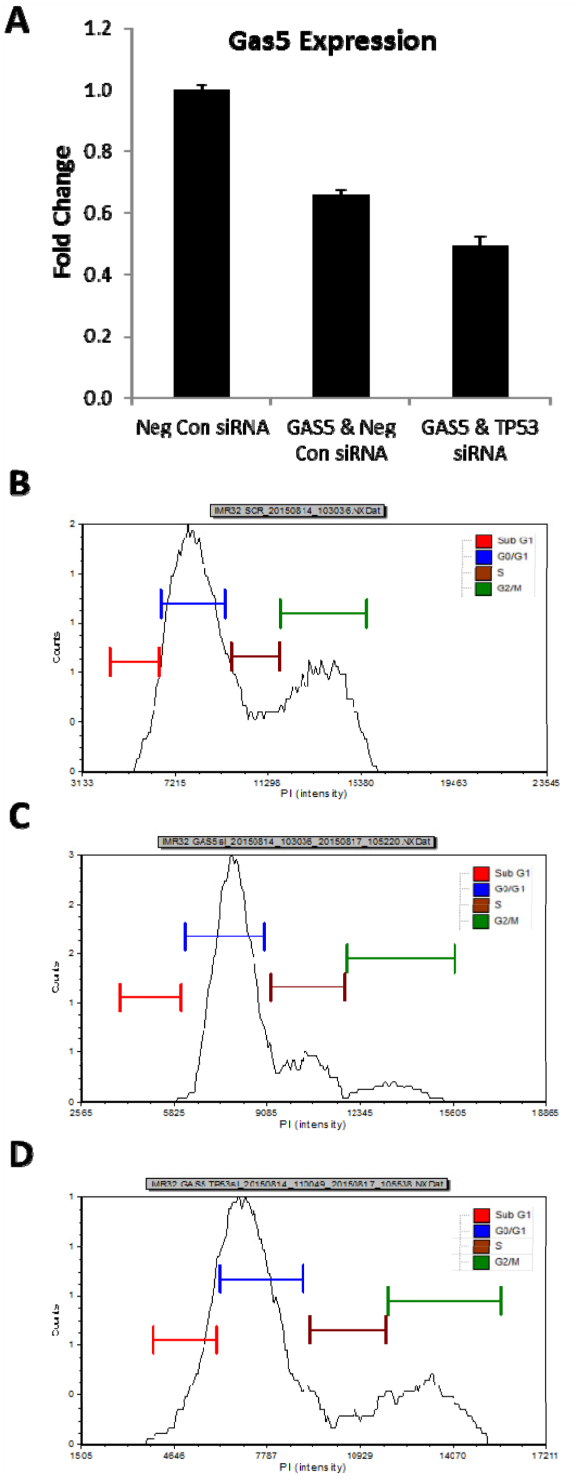

B

Cell Population	% of Gated Cells	CV
Total	100 %	
Sub G1	3.0 %	2.2 %
G0/G1	51.0 %	8.9 %
S	10.0 %	5.4 %
G2/M	30.0 %	7.0 %

C

Cell Population	% of Gated Cells	CV
Total	100 %	
Sub G1	0.0 %	0.0 %
G0/G1	72.6 %	5.8 %
S	16.8 %	6.2 %
G2/M	7.4 %	5.0 %

D

Cell Population	% of Gated Cells	CV
Total	100 %	
Sub G1	11.4 %	8.6 %
G0/G1	60.8 %	10.3 %
S	7.6 %	6.7 %
G2/M	17.7 %	5.9 %

Supplementary Figure 10: Effect of the knock-down of p53 and GAS5 in IMR-32 cells as measured by the Cell Cycle. **A.** Confirmation of the knock-down of GAS5 siRNA into IMR-32 cells by qRT-PCR. Samples were normalized to the Ct value of GAPDH and expressed as a means of +/- SD using three biological replicates. Individual traces of transfected cells after propidium iodine staining: **B.** Negative Control cell populations indicated 3.0% in Sub G1, 51.0% in G0/G1, 10.0% in S phase, and 30.0% in G2/M, **C.** Co-transfected GAS5 & Negative Control siRNA cell populations indicated 0.0% in Sub G1, 72.6% in G0/G1, 16.8% in S phase, and 7.4% in G2/M, and **D.** Co-transfected p53 & GAS5 siRNA cell populations indicated 11.4% in Sub G1, 60.8% in G0/G1, 7.6% in S phase, and 17.7% in G2/M. All experiments were performed in triplicate.

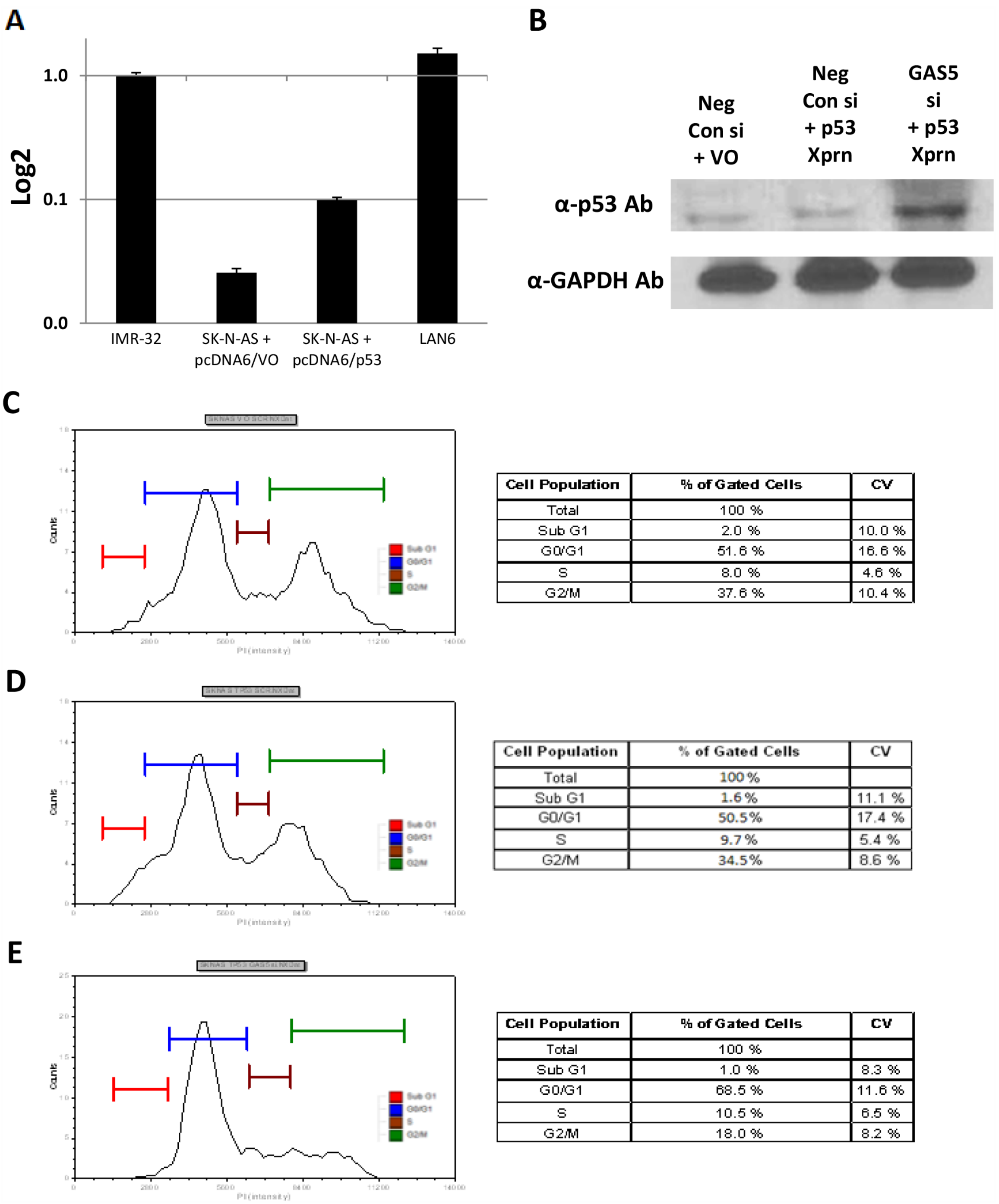

C

Cell Population	% of Gated Cells	CV
Total	100 %	
Sub G1	2.0 %	10.0 %
G0/G1	51.6 %	16.6 %
S	8.0 %	4.6 %
G2/M	37.6 %	10.4 %

D

Cell Population	% of Gated Cells	CV
Total	100 %	
Sub G1	1.6 %	11.1 %
G0/G1	50.5 %	17.4 %
S	9.7 %	5.4 %
G2/M	34.5 %	8.6 %

E

Cell Population	% of Gated Cells	CV
Total	100 %	
Sub G1	1.0 %	8.3 %
G0/G1	68.5 %	11.6 %
S	10.5 %	6.5 %
G2/M	18.0 %	8.2 %

Supplementary Figure 11: Effect of complementary exogenous expression of p53 and knock-down of GAS5 in SK-N-AS cells. A. Confirmation of the exogenous expression of p53 via plasmid transfection (pcDNA6/p53) into SK-N-AS cells by qRT-PCR compared to vector control cells, IMR-32, and LA-N-6 cells. Samples were normalized to the Ct value of GAPDH and expressed as a means of +/- SD using three biological replicates. **B.** Western blot analysis of p53 protein expression in SK-N-AS cells after transfection with Negative Control siRNA + Vector only, Negative Control siRNA + p53 expression plasmid, and GAS5 siRNA + p53 expression plasmid. GAPDH was used as a load control. **C, D,** and **E.** Individual traces of transfected cells after propidium iodine staining: C) "Vector only" + Negative Control siRNA cell populations 51.6% in G0/G1, 8.0% in S phase, and 37.6% in G2/M, D) p53 expression plasmid + Negative Control siRNA cell populations indicated 50.5% in G0/G1, 9.7% in S phase, and 34.5% in G2/M, and E) p53 expression plasmid + GAS5 siRNA cell populations indicated 68.5% in G0/G1, 10.5% in S phase, and 18.0% in G2/M. All experiments were performed in triplicate.

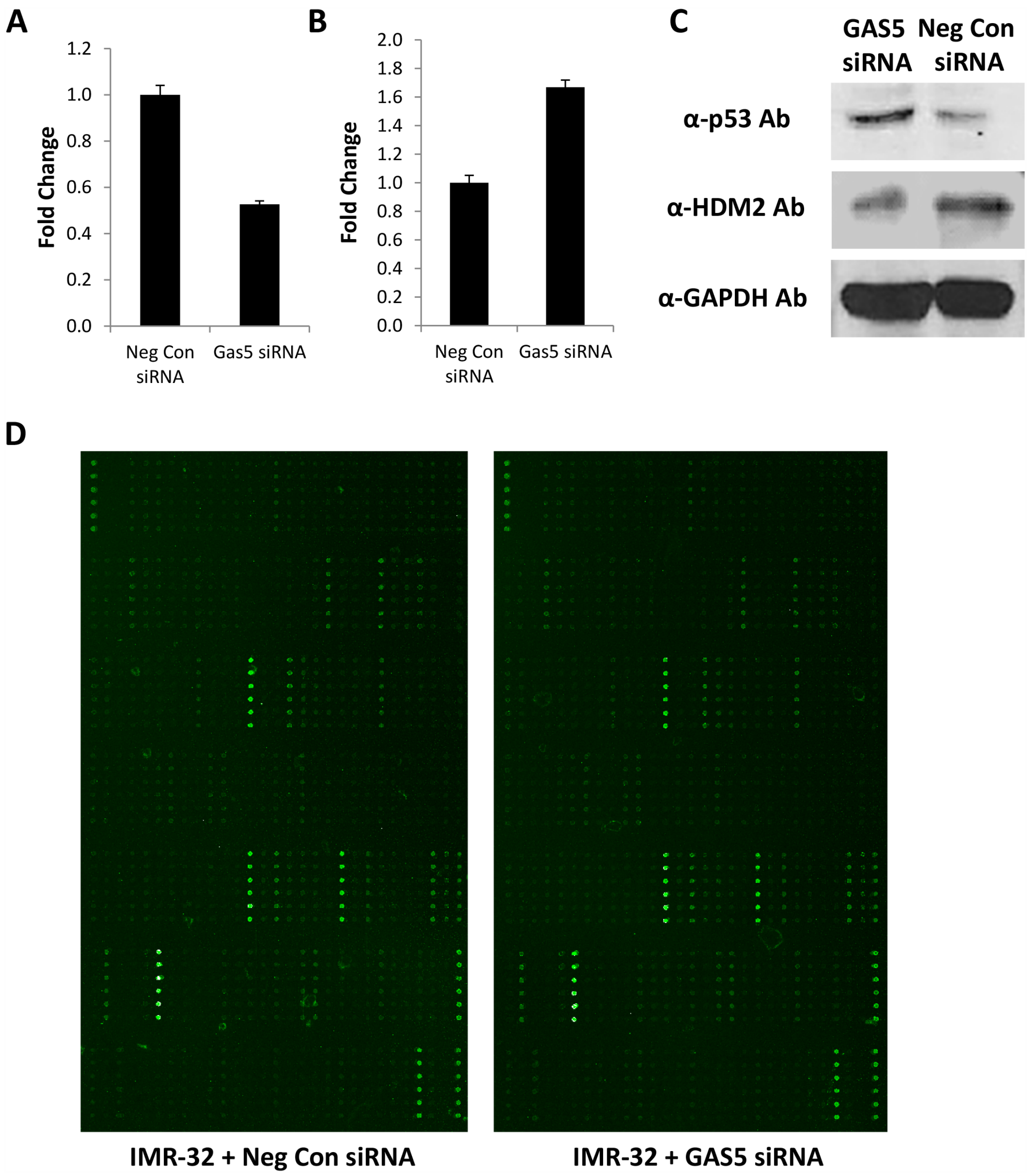

Supplementary Figure 12: Analysis of GAS5 knock-down in IMR-32 cells for use in p53 phospho antibody microarray. **A.** Confirmation of the knock-down of GAS5 by siRNA in IMR-32 cells by qRT-PCR. **B.** Confirmation of the expression of p53 after knock-down with GAS5 siRNA in IMR-32 cells by qRT-PCR. Samples were normalized to the Ct value of GAPDH and expressed as a means of +/- SD using three biological replicates. Samples were normalized to the Ct value of GAPDH and expressed as a means of +/- SD using three biological replicates. **C.** Western blot analysis of p53 and HDM2 (MDM2) in IMR-32 cells after transfection with Negative Control siRNA vs GAS5 siRNA. GAPDH was used as a load control. **D.** Scan of p53 phosphorylation antibody microarrays. Cell lysates used were screened (as above in S12A, S10B, & S10C) prior to use. Each spot on the array is scanned and measured with 6 replicates.

Ratio Analysis

Antibody List		Signal Ratios		Ratio Changes
		4000016013 (C)	4000016012 (T)	T/C
BRCA1 (Phospho-Ser1423)	BRCA1 (Ab-1423)	0.12	0.14	1.18
BRCA1 (Phospho-Ser1457)	BRCA1 (Ab-1457)	0.82	1.17	1.43
BRCA1 (Phospho-Ser1524)	BRCA1 (Ab-1524)	0.72	0.72	1.01
p53 (Phospho-Ser15)	p53 (Ab-15)	1.51	1.29	0.85
p53 (Phospho-Thr18)	p53 (Ab-18)	1.22	0.95	0.78
p53 (Phospho-Ser20)	p53 (Ab-20)	0.46	0.53	1.17
p53 (Phospho-Ser315)	p53 (Ab-315)	0.28	0.39	1.35
p53 (Phospho-Ser33)	p53 (Ab-33)	1.05	0.88	0.84
p53 (Phospho-Ser37)	p53 (Ab-37)	1.12	0.94	0.84
p53 (Phospho-Ser378)	p53 (Ab-378)	1.27	1.64	1.30
p53 (Phospho-Ser392)	p53 (Ab-392)	0.88	1.01	1.15
p53 (Phospho-Ser46)	p53 (Ab-46)	2.78	2.97	1.07
p53 (Phospho-Ser6)	p53 (Ab-6)	1.50	1.44	0.96
p53 (Phospho-Ser9)	p53 (Ab-9)	1.03	1.03	1.00

Min	0.62
Max	1.43
Mean	1.02

Supplementary Figure 13: Analysis of p53 phospho antibody microarray. Analysis was performed by measuring median signal intensity extracted from the array image, determining the average signal intensity of replicate spots, then determining the median value of the Average Signal Intensity for all antibodies within each array slide. This normalization was used to determine the fold change between the negative control siRNA and GAS5 siRNA treatment samples. The Ratio Changes were determined by measuring the Signal Intensity of a Phospho Site-Specific Antibody divided by the Signal Intensity of the Site-Specific Antibody for each treatment, then dividing the ratio of the GAS5 siRNA treatment samples by the ratio of the negative control siRNA. The results are highlighted in different shades of red and green: *Red* = increase in expression, *Green*: = decrease in expression. The results highlighted in yellow represent the best candidates indicated by the array.

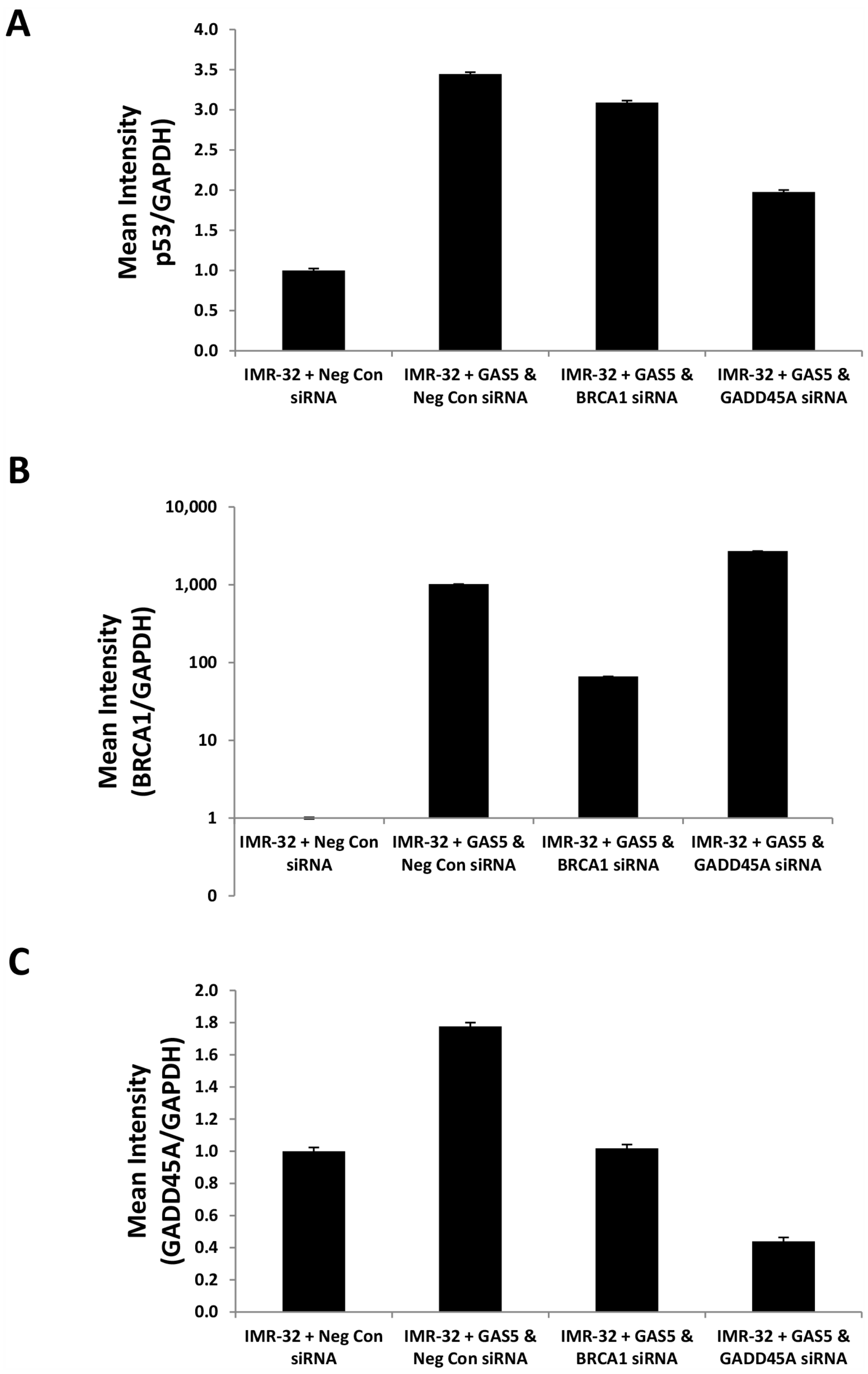

Supplementary Figure 14: Quantitation of Western blot analysis of p53, BRCA1-phos1457, and GADD45A after GAS5, BRCA1, and GADD45A knock-down. Scanning and quantitation for all bands performed using NIS-Elements AR Analysis 4.2 software with data normalized to the Mean Intensity of the appropriate GAPDH load control. Graphs represent analysis of: **A.** p53, **B.** BRCA1-phos1457, and **C.** GADD45A based upon blot represented in Figure 6B.

A

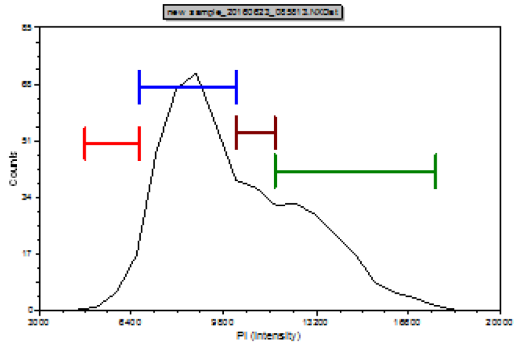

Cell Population	%of Gated Cells	CV	Concentration
Total	100 %		0.44 x 10^6 cells/mL
Sub G1	2.0 %	4.7 %	0.01 x 10^6 cells/mL
G0/G1	56.2 %	9.9 %	0.25 x 10^6 cells/mL
S	9.5 %	4.1 %	0.06 x 10^6 cells/mL
G2/M	28.8 %	10.1 %	0.13 x 10^6 cells/mL

B

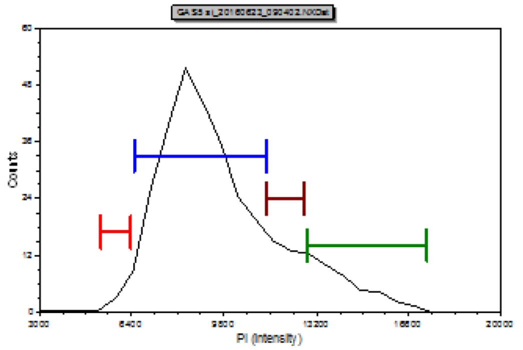

Cell Population	% of Gated Cells	CV	Concentration
Total	100 %		0.33 x 10^6 cells/mL
Sub G1	0.3 %	7.5 %	0.00 x 10^6 cells/mL
G0/G1	77.2 %	12.8 %	0.26 x 10^6 cells/mL
S	16.8 %	3.1 %	0.03 x 10^6 cells/mL
G2/M	7.7 %	6.7 %	0.04 x 10^6 cells/mL

C

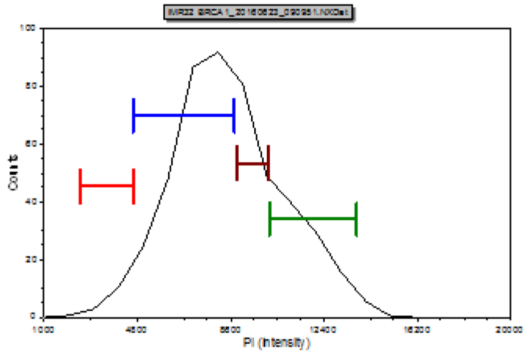

Cell Population	% of Gated Cells	CV	Concentration
Total	100 %		0.49 x 10^6 cells/mL
Sub G1	2.3 %	12.2 %	0.01 x 10^6 cells/mL
G0/G1	60.2 %	13.5 %	0.30 x 10^6 cells/mL
S	7.6 %	3.7 %	0.07 x 10^6 cells/mL
G2/M	13.8 %	7.8 %	0.11 x 10^6 cells/mL

D

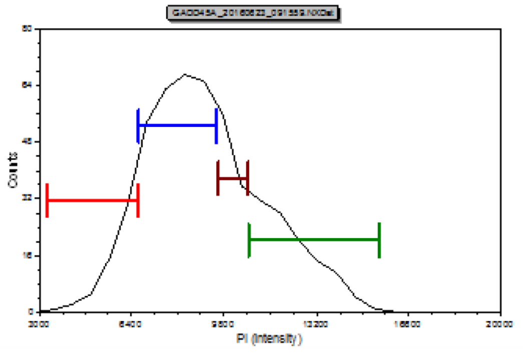

Cell Population	%of Gated Cells	CV	Concentration
Total	100 %		0.46 x 10^6 cells/mL
Sub G1	4.9 %	9.0 %	0.02 x 10^6 cells/mL
G0/G1	59.7 %	8.6 %	0.28 x 10^6 cells/mL
S	10.5 %	2.9 %	0.05 x 10^6 cells/mL
G2/M	22.3 %	7.8 %	0.10 x 10^6 cells/mL

Supplementary Figure 15: Effect of simultaneous GAS5 and BRCA1 or GADD45A knock-down on the Cell Cycle. **A.** IMR-32 cells transfected with Negative Control siRNA, **B.** IMR-32 cells transfected with GAS5 siRNA, **C.** IMR-32 cells transfected with GAS5 and BRCA1 siRNA, **D.** IMR-32 cells transfected with GAS5 and GADD45A siRNA. All samples were stained with propidium iodine and cell populations were then measured in Sub G1, G0/G1, S phase, and G2/M. All experiments were performed in triplicate.

A

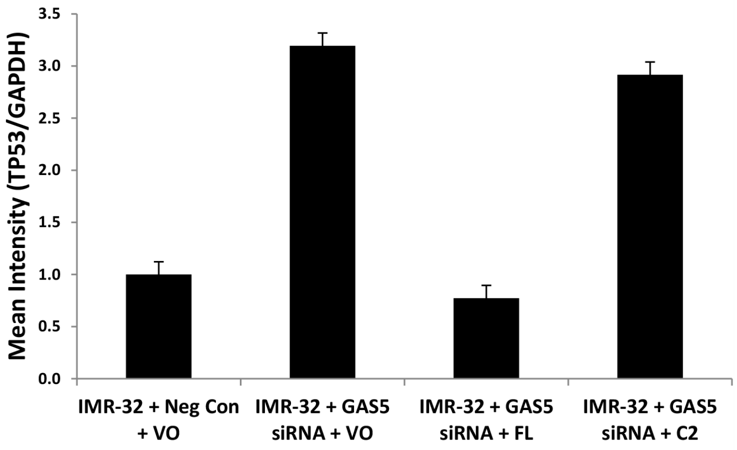

B

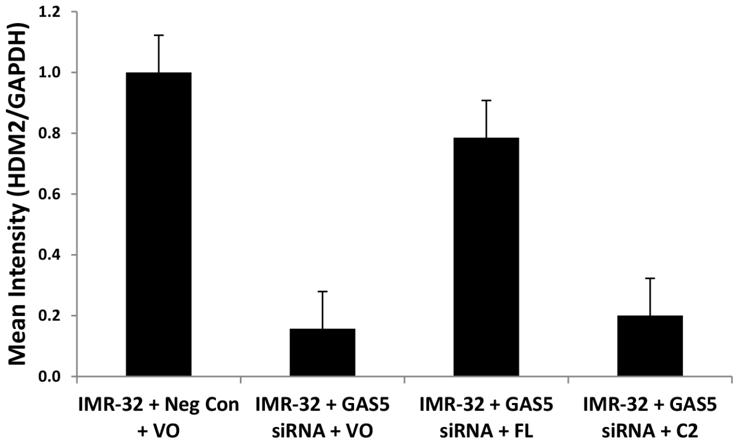

Supplementary Figure 16: Quantitation of Western blot analysis of p53 and HDM2 after GAS5 knock-down and complementation with FL & C2 variants. Scanning and quantitation for all bands performed using NIS-Elements AR Analysis 4.2 software with data normalized to the Mean Intensity of the appropriate GAPDH load control. Graphs represent analysis of: **A.** p53 and **B.** HDM2 based upon blot represented in Figure 7B.

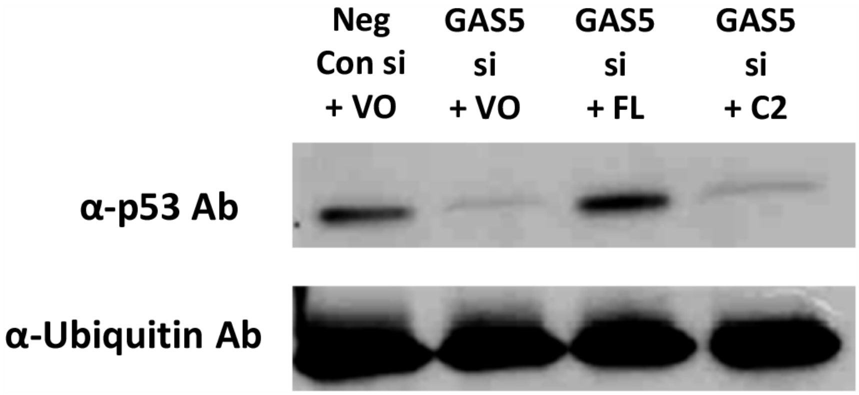

Supplementary Figure 17: Examination of the Ubiquitination of p53 after GAS5 knock-down and complementation with FL & C2 variants. Western blot analysis of Ubiquitin and p53 after immunoprecipitation of Ubiquitin in IMR-32 cells transfected as follows: Negative Control siRNA & Vector only (VO), GAS5 siRNA + VO, GAS5 siRNA complemented with FL-expressing plasmid, and GAS5 siRNA complemented with C2-expressing plasmid.
